# Diagnostic performance enhancement of pancreatic cancer using proteomic multimarker panel

**DOI:** 10.18632/oncotarget.21861

**Published:** 2017-10-16

**Authors:** Jiyoung Park, Yonghwan Choi, Junghyun Namkung, Sung Gon Yi, Hyunsoo Kim, Jiyoung Yu, Yongkang Kim, Min-Seok Kwon, Wooil Kwon, Do-Youn Oh, Sun-Whe Kim, Seung-Yong Jeong, Wonshik Han, Kyu Eun Lee, Jin Seok Heo, Joon Oh Park, Joo Kyung Park, Song Cheol Kim, Chang Moo Kang, Woo Jin Lee, Seungyeoun Lee, Sangjo Han, Taesung Park, Jin-Young Jang, Youngsoo Kim

**Affiliations:** ^1^ Department of Biomedical Sciences, Seoul National University College of Medicine, Seoul, Korea; ^2^ Department of Biomedical Engineering, Seoul National University College of Medicine, Seoul, Korea; ^3^ Immunodiagnostics R&D Team, IVD Business Unit 5, SK Telecom, Seoul, Korea; ^4^ Department of Statistics, Seoul National University, Seoul, Korea; ^5^ Department of Surgery and Cancer Research Institute, Seoul National University College of Medicine, Seoul, Korea; ^6^ Department of Internal Medicine and Cancer Research Institute, Seoul National University Hospital, Seoul, Korea; ^7^ Department of Surgery, Samsung Medical Center, Sungkyunkwan University School of Medicine, Seoul, Korea; ^8^ Internal Medicine, Samsung Medical Center, Sungkyunkwan University School of Medicine, Seoul, Korea; ^9^ Department of Internal Medicine, Seoul National University Hospital Healthcare System Gangnam Center, Seoul, Korea; ^10^ Department of Surgery, University of Ulsan College of Medicine and Asan Medical Center, Seoul, Korea; ^11^ Department of Surgery, Severance Hospital, Yonsei University College of Medicine, Seoul, Korea; ^12^ Center for Liver Cancer, National Cancer Center, Seoul, Korea; ^13^ Department of Mathematics and Statistics, Sejong University, Seoul, Korea

**Keywords:** pancreatic cancer, proteomics, diagnostic biomarker, multimarker panel, multiple reaction monitoring

## Abstract

Due to its high mortality rate and asymptomatic nature, early detection rates of pancreatic ductal adenocarcinoma (PDAC) remain poor.

We measured 1000 biomarker candidates in 134 clinical plasma samples by multiple reaction monitoring-mass spectrometry (MRM-MS). Differentially abundant proteins were assembled into a multimarker panel from a training set (n=684) and validated in independent set (n=318) from five centers. The level of panel proteins was also confirmed by immunoassays. The panel including leucine-rich alpha-2 glycoprotein (LRG1), transthyretin (TTR), and CA19-9 had a sensitivity of 82.5% and a specificity of 92.1%. The triple-marker panel exceeded the diagnostic performance of CA19-9 by more than 10% (AUC_CA19-9_ = 0.826, AUC_panel_= 0.931, P < 0.01) in all PDAC samples and by more than 30% (AUC_CA19-9_ = 0.520, AUC_panel_ = 0.830, P < 0.001) in patients with normal range of CA19-9 (<37U/mL). Further, it differentiated PDAC from benign pancreatic disease (AUC_CA19-9_ = 0.812, AUC_panel_ = 0.892, P < 0.01) and other cancers (AUC_CA19-9_ = 0.796, AUC_panel_ = 0.899, P < 0.001).

Overall, the multimarker panel that we have developed and validated in large-scale samples by MRM-MS and immunoassay has clinical applicability in the early detection of PDAC.

## INTRODUCTION

Pancreatic ductal adenocarcinoma (PDAC) is one of the most lethal gastrointestinal malignancies and the seventh leading cause of cancer-related deaths. Most pancreatic cancer (PC) patients die within 1 year after the initial diagnosis, and approximately 7% survives over 5 years. However, the absence of symptoms in its initial stages and insufficient early detection tools lead to poor prognoses, and roughly 80% of the disease is unresectable at the time of diagnosis [1]. The close evaluation is limited to symptomatic or recurrent cancer patients in addition to lack of cost-effective, specific, and reliable screening tests [2]. Current diagnostic tools (e.g., imaging, biopsy) are likely to be expensive, time-consuming, and invasive. Thus, there is an unmet need for a clinical examination method that can discriminate malignancy from normal and benign states.

The identification of a biologically derived indicator is imperative for diseases without an appropriate treatment and those that experience rapid progression, leading to high mortality, because regular screening lowers the rates of late detection. Distant metastases are a contraindication to pancreatic resection, which is the only available definitive treatment [3]. The lack of accessibility to general screening contributes to the inability to detect PC, which allows the metastasis to progress and decreases survival rates by 50%.

The only biomarker for pancreatic cancer (PC) that is clinically approved by the US Food and Drug Administration (FDA) is serum CA-19-9. Unfortunately, despite its approximately 79% sensitivity and 82% specificity, CA-19-9 is inadequate for the early detection of PC in asymptomatic patients, and there is no individual marker that diagnoses PC with satisfactory sensitivity and specificity. Further, 10% to 15% of PC patients do not express CA19-9 due to their lack of Lewis A antigen [4]. Other benign diseases (e.g., obstructive jaundice) also increase CA19-9 levels [5]. Thus, CA19-9 merely indicates recurrence or functions as an ancillary modality to imaging devices, such as computed tomography (CT) and endoscopic ultrasound (EUS), in the primary diagnosis of PC [6].

Whereas many potential biomarkers have been suggested, the integration of unstructured data and their validation are insufficient [7]. Thus, translation of these compounds to the clinic has been difficult. Many reports have addressed the problems of invalid results from small sample sizes, the complexity of samples with various dynamic ranges of analytes, and the lack of a comprehensive biomarker development pipeline [8, 9]. To this end, we performed a large-scale multicenter validation of a multimarker panel by multiple reaction monitoring-mass spectrometry (MRM-MS) and antibody-based assays to measure the levels of established and newly discovered biomarkers in PC patients and control subjects.

Usually, biomarker discovery provides myriad candidate proteins that need to be verified in various types of patient samples. Thus, MRM-MS, a highly selective and sensitive method of quantitating targeted proteins or peptides in samples, is a possible alternative to current PC screens [10]. Compared to typical antibody-based clinical assays, MRM-MS is a targeted proteomics technology that measures at least 100 protein targets per sample simultaneously with precision [11]. Further, the existence of the antibodies is irrelevant to the quantitation in the validation of a large number of samples. In addition, MRM-MS generates consistent and reproducible datasets from highly complex samples between laboratories [12]. Thus, there is no bottleneck from the discovery to validation phase of most potential biomarkers in a single platform. We exploited the high-throughput MRM-MS assay to discover many potential targets and validated the results by a conventional antibody-based method to ensure that the new technology generated reliable data and that the identified markers could be translated easily into clinical practice.

To construct a better panel for screening PC, we measured the levels of differentially expressed proteins in various groups—healthy people and patients with benign diseases, PC, and other cancers. The resulting triple-marker panel (LRG1, TTR, and CA19-9) was analyzed statistically to correlate the MRM-MS and antibody-based assay data with disease status and predict malignancy without perceptible signs. The panel performed significantly better than CA19-9 in different conditions. Thus, we report the efficacy of our panel by large-scale multicenter assessment of previously reported and newly discovered biomarkers.

## RESULTS

### Study population

The study participants were recruited from 5 major medical centers in Seoul, Korea: National Cancer Center (NCC), Seoul National University Hospital or Seoul National University Hospital Healthcare System Gangnam Center (SNUH), Samsung Medical Center (SMC), Asan Medical Center (AMC), and Yonsei Severance Hospital (YSH). For the discovery and verification study, 134 plasma samples [pancreatic ductal adenocarcinoma (PDAC) =50, pancreatic benign (PB) =34, normal control (NL) =50] were drawn between January 2011 and December 2013. All samples, except for PDAC, were acquired from our study on intraductal papillary mucinous neoplasms (IPMNs) [13], the purpose of which differed and in which the data were processed independently. The details on the collection of clinical samples are provided on Supplementary Materials. For the cross-platform validation studies, 1008 plasma samples, including those that were used in the verification step [PDAC =401, NL =349, other cancer (OC) =149, PB =109], were collected between January 2011 and December 2013. The normal control group comprised a healthy population without any malignancies or other serious health conditions and individuals with benign diseases, such as gallbladder stones and cholecystitis without severe inflammation. The benign pancreatic disease group was composed of patients with intraductal papillary mucinous neoplasm (IPMN). Tumor stages were classified per the 7^th^ edition of AJCC [14]. The clinicopathological data on the study subjects are summarized in Supplementary Table 1 for the verification study and Table 1 for the validation study.

**Table 1:** Demographics of validation study population

Group			PDAC			NL	OC		PB		
Institute	NCC	AMC	SNUH	YSH	SMC	SNUH	SNUH	YSH	AMC	SMC	SNU
N total	128	75	50	47	101	349	149	27	47	30	5
Age mean (SD)	62.94 (9.81)	62.2 (10.59)	59.44 (9.34)	64.6 (8.56)	59.76 (11.48)	56.94 (8.07)	55.22 (11.51)	59.96 (14.15)	50.6 (12.86)	49.99 (15.22)	55.2 (8.93)
BMI mean (SD)	23.32 (2.88)	22.94 (3.23)	22.4 (3.32)	23.13 (2.85)	22.52 (2.95)	23.84 (3.06)	23.44 (3.41)	23.69 (3.01)	23.26 (4.71)	22.89 (3.04)	23.38 (2.08)
Sex ratio (Male %)	65.63	61.33	56	61.7	63.37	55.87	25.5	51.85	34.04	33.33	80
Alcohol ratio (# missing)	50.78 (0)	52 (0)	14.58 (2)	38.3 (0)	37.37 (2)	79.08 (61)	22.3 (1)	40.74 (0)	42.55 (0)	26.67 (0)	20 (0)
Smoking ratio (# missing)	49.22 (0)	38.67 (0)	18.75 (2)	36.17 (0)	44.58 (18)	41.55 (71)	8.11 (1)	37.04 (0)	34.04 (0)	25 (10)	40 (0)
CA19-9 median	312.5	51.7	82.85	59.3	363.45	7.4	9.5	7.5	7	9.33	11
CA19-9 MAD	8032.5	415.05	358.68	328.9	1531.29	3.6	12.5	9.55	17.3	8.38	27.9
CA19-9 missing	0	0	0	0	1	0	104	1	0	1	0
CA19-9 censor	12	4	0	0	0	38	0	0	0	0	1
CEA median	5.35	2.3	1.8	2.73	2.52	1.3	1.7	2	1.1	0.79	1.7
CEA MAD	17.35	2.675	1.225	4.055	2.93	0.4	1.4	3	1.315	0.87	1.4
CEA missing	0	1	2	0	56	0	58	2	0	9	0
CEA censor	0	0	1	0	0	37	0	0	0	0	0
Stage of cancer						Breast (n=52)	Colon (n=45)	Thyroid (n=52)			
I	3	10	3	3	1	19	10	22			
II	27	63	45	43	50	28	12	1			
III	25	1	2	1	2	3	14	29			
IV	73	1	0	0	48	0	9	0			

PC, pancreatic cancer; NL, normal; OC, other cancer (thyroid, colon, and breast); PB, pancreatic benign (pancreatitis, IPMN, neuroendocrine tumor, solid pseudopapillary neoplasm, mucinous cystic neoplasm, serous cystadenoma, pseudocyst, pancreatolithiasis); SD, standard deviation; NCC, National Cancer Center; AMC: Asan Medical Center; SNU, Seoul National University; YSH, Yonsei Severance Hospital; SMC, Samsung Medical Center; CA19-9, carbohydrate antigen 19-9; CEA, carcinoembryonic antigen; MAD, median absolute deviation.

The study protocols were approved by the corresponding institutional review boards of all participating institutions (SNUH surgery H-0901-010-267, SNUH internal medicine H-0412-138-005 and H-0412-138-006, SNUH HSGC H-1305-573-489 and C-1301-095-458, YSH 4-2013-0725, NCC NCCNCS13818, SMC 2008-07-065, AMC 2013-1061), and informed consent was obtained from all participants who contributed biospecimens.

### Selection of PC-related candidate biomarkers

**Figure 1:**
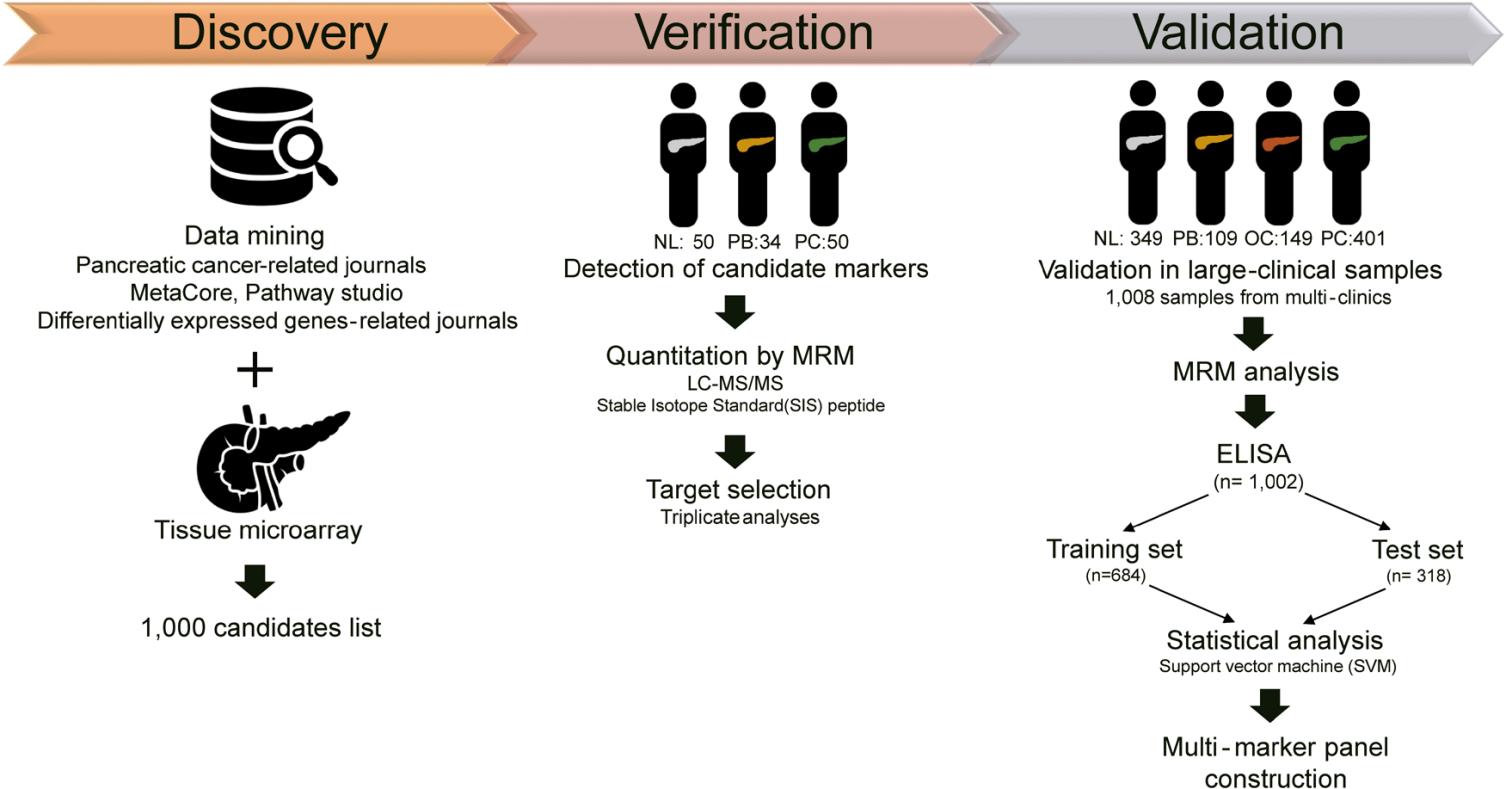
Overall scheme of the study Through literature searches of pancreatic cancer-related journals, public databases, and journals on differentially expressed genes, in addition to microarray data from a previous study [32], 1000 candidates were identified. Then, the potential markers were detected in 134 samples, composed of 50 normal controls (25 normal and 25 benign status, such as cholecystitis), 34 pancreatic benign disease (IPMN), and 50 PDAC groups. Targets were narrowed down by LC-MS/MS assay with stable isotope standard (SIS) peptide. A total of 54 proteins, or 68 peptides, were validated in a large clinical sample [n=1008; 349 normal, 109 pancreatic benign diseases, 149 other cancer (thyroid, breast, and colorectal cancer), and 401 pancreatic cancer] by MRM analysis and ELISA [n=1002; 348 normal, 109 pancreatic benign diseases, 149 other cancer (thyroid, breast, and colorectal cancer), and 396 pancreatic cancer]. The multimarker panel was ultimately constructed by statistical analysis and supporting vector machine (SVM) method.

Candidate markers were chosen from an extensive database and literature search, which generated 508 proteins that were related to PC, 22 traditional cancer markers, and 14 known mutated proteins [15–31]. From the previous microarray analysis of resected PC tissue, 456 proteins were identified [32] (Supplementary Figure 1 and Supplementary Materials). Of these 1000 initial candidates, the following criteria were used to select candidates (Table 2 and Supplementary Table 2): (i) 907 proteins were filtered based on MS/MS spectra from the National Institute of Standards and Technology (NIST) MS/MS library for empirical evidence of target detection by mass spectrometry. (ii) Of the 907 proteins, 225 were actually detected in pooled plasma samples. (iii) Based on relative quantitation against an internal standard (β-galactosidase), 205 proteins and 316 peptides had AUC > 0.60 between the NL, PB, and PC groups. To select true positives, SRM collider and BLAST were performed to analyze unique transitions and peptides, respectively. (iv) Detected in biological samples by unique peptides, 217 SIS (stable isotope-labeled standard) peptides, which represented 176 proteins, were synthesized and quantified in 134 samples. Consequently, 79 peptides (65 proteins) were differentially quantified. (v) Relative quantification of 65 proteins and 79 peptides with SIS peptides in triplicate analyses resulted in 54 proteins and 68 peptides that had AUC > 0.60 between the NL and PC groups (Supplementary Table 3). Then, the automated detection of inaccurate and imprecise (AuDIT) algorithm was used to improve the selection of true positive targets by measuring the coefficient of variation (CV) in multiple analyses and comparing the relative intensities of analytes and SIS peptides to remove the possible interferences [33]. (vi) Finally, 68 interference-free peptides from 54 proteins that were confidently verified were applied to 1008 plasma samples using the MRM-MS platform. The overall scheme of the study is summarized in Figure 1 and Table 2. Collectively, the systematic selection of candidate targets was feasible in the plasma samples.

**Table 2:** Refinement of the 1000 candidates down to the 3-protein panel

Process		Number of proteins^a^	Refinement	Methodology	Clinical samples
**Discovery**	**1000**	508	PC-relevant proteins	Database and literature search	-
		456		Microarray analysis	Tissue samples(n=173)
		22		Traditional cancer markers	-
		14		Known mutated proteins	-
	**205**		Single-marker analysis	MRM-MS (w/β-galactosidase)	Single-center case-control blank plasma samples (n=134)
	**176**			MRM-MS (w/SIS peptides)	
**Verification**	**65**		Triplicate analysis	MRM-MS (w/SIS peptides)	
**Validation**	**54**		Differential single-marker candidates	MRM-MS (w/SIS peptides)	Multicenter case-control blank plasma samples (n=1,008)
	**5**		5 panels selected for immunoassay	Multimarker analysis	
	**3**		1 panel tested	Immunoassay	

^a^ the number of input proteins.

### Confirmation of promising markers in the training/validation set by MRM-MS

In blood-based diagnostic methods, markers must perform reproducibly in a clinical environment. To confirm this property, we applied 3 criteria with regard to the stability of detection in the MRM-MS experiments and obtained 68 peptides, or 54 proteins (Supplementary Figure 2):

1. Coefficient of variance (CV) in triplicate analyses of the verification. Applying a cutoff of 10% CV, 26 peptides (22 proteins) were determined to be unstable.
2. Relative peptide level (endogenous:SIS peptide ratio). To quantify proteins, we chose representative transitions that provided the highest measurement as the signature peptide, for which the reference range was 0.1 < (relative peptide level) < 10. There were 12 peptides (11 proteins) that did not lie in this range and were excluded from the analysis.
3. Confounding factors, including clinical centers and bias across batches in the enrichment step by MRMMS liquid chromatography (LC). A total of 14 peptides (10 proteins) appeared to be affected by these factors and were thus excluded.

Consequently, 34 peptides (25 proteins) and CA19-9 were selected for further analysis. Candidates were considered for all combinations of 1 to 5 biomarkers. We observed that the combinations of 2 or 3 markers resulted in approximately 90% specificity and 80% sensitivity, but the addition of more markers did not alter the performance significantly. Thus, we chose the classifier with 3 biomarkers as a final candidate. The performance of each combination (2^25+1^ ≈ 64,000,000 protein combinations) of CA19-9 and other two proteins was tested by support vector machine (SVM) in a training set (case n=316, control n=368) and a test set 1 (case n=80, control n=89) (Supplementary Table 4). We measured AUC values and sensitivity at a specificity of 90% (Sn|_Sp=0.9_) to select a marker or sets of markers that outperformed CA19-9 alone. From the 5-fold cross validation of the training and test sets, we developed 18 panels, which resulted in at least a 7% higher AUC value than CA19-9 (p-value<0.05, DeLong's test) [34]. Then, we selected 6 panels with sensitivity over 10% greater than that of CA19-9 when the specificity was fixed to 0.90 (p-value<0.05, McNemar's test). Ultimately, 5 triple-marker panels that satisfied all criteria with regard to AUC values and sensitivity were selected (Supplementary Table 5). To examine reproducible performance across platforms from MRM-MS and immunoassay, we selected a panel, comprising LRG1, TTR, and CA19-9, by considering their relevance to tumorigenesis, relatively high protein concentration in blood, and availability in commercial immunoassays. The linear response curve of these peptides is presented in Supplementary Figure 3. Taken together, MRM-MS assays and immunoassays were developed for reproducible markers in clinical samples.

### Diagnostic performance of triple-marker panel in early detection

To validate the relative quantitation of the candidate proteins, we recorded the levels of natural proteins by immunoassays. Immunoassay 1 measured LRG1 by enzyme-linked immunosorbent assay (ELISA) and TTR by immunoturbidimetric assay (ITA), whereas Immunoassay 2 measured both LRG1 and TTR by ELISA. Because the results of the two assays did not significantly alter the outcomes, we presented the data by ELISA (Immunoassay 2) throughout the paper but also provided the Immunoassay 1 findings in Table 3.

**Table 3:** Performance of triple-marker panel vs. CA19-9

	Performancecomparison	CA19-9	CA19-9 + LRG1 + TTR		
			MRM-MS	Immunoassay 1	Immunoassay 2
**Control vs. PDAC**	AUC	0.826	0.931 (11% ↑) ^**^	0.940 (11% ↑) ^***^	0.932 (11% ↑) ^***^
	Specificity	0.888	0.921	0.899	0.944
	Sensitivity	0.725	0.825 (10% ↑)	0.825 (10% ↑)	0.825 (10% ↑)
**Control vs. PDACStage I & II**	AUC	0.792	0.907 (11% ↑) ^**^	0.915 (12% ↑) ^**^	0.914 (12% ↑) ^**^
	Specificity	0.888	0.921	0.899	0.944
	Sensitivity	0.640	0.760 (12% ↑)	0.760 (12% ↑)	0.780 (14% ↑)
**Other Cancervs. PDAC**	AUC	0.796	0.899 (10% ↑) ^***^	0.897 (10% ↑) ^**^	0.898 (10% ↑) ^***^
	Specificity	0.879	0.839	0.826	0.866
	Sensitivity	0.725	0.825 (10% ↑) ^**^	0.825 (10% ↑) ^**^	0.825 (10% ↑) ^*^
**Pancreatic Benignvs. PDAC**	AUC	0.812	0.892 (8.0% ↑)	0.898 (8.6% ↑) ^*^	0.895 (8.3% ↑) ^**^
	Specificity	0.810	0.857	0.810	0.857
	Sensitivity	0.725	0.825 (10% ↑)	0.825 (10% ↑) ^*^	0.825 (10% ↑)
**Control vs. PDAC^+^**	AUC	0.520	0.830 (31% ↑) ^***^	0.835 (32% ↑) ^***^	0.829 (31% ↑) ^***^
	Specificity	0.888	0.921	0.899	0.944
	Sensitivity	0.241	0.517 (28% ↑)	0.517 (28% ↑)	0.517 (28% ↑)
**Control vs. PDACStage I & II^+^**	AUC	0.567	0.818	0.832	0.834
**Other Cancer vs.PDAC^+^**	AUC	0.519	0.767	0.741	0.726
**Pancreatic Benign vs.PDAC^+^**	AUC	0.520	0.829	0.829	0.830

Performance of the panel was assessed by several assays in 5 test sets as described in Supplementary Table 4. The sensitivity and specificity were obtained by applying the cutoff value from the training set when the specificity was fixed to 0.9. DeLong's test was used to evaluate AUC values, and two-sided p-values lower than 0.05 were considered to be significant.

^*^ DeLong's test, p-value < 0.05, ^**^ DeLong's test, p-value < 0.01, ^***^ DeLong's test, p-value < 0.001. + PDAC samples with the normal range of CA19-9 (<37U/mL).

Immunoassay 1: LRG1 measured by ELISA, TTR measured by immunoturbidimetric assay (ITA); Immunoassay 2: LRG1 and TTR both measured by ELISA.

**Figure 2:**
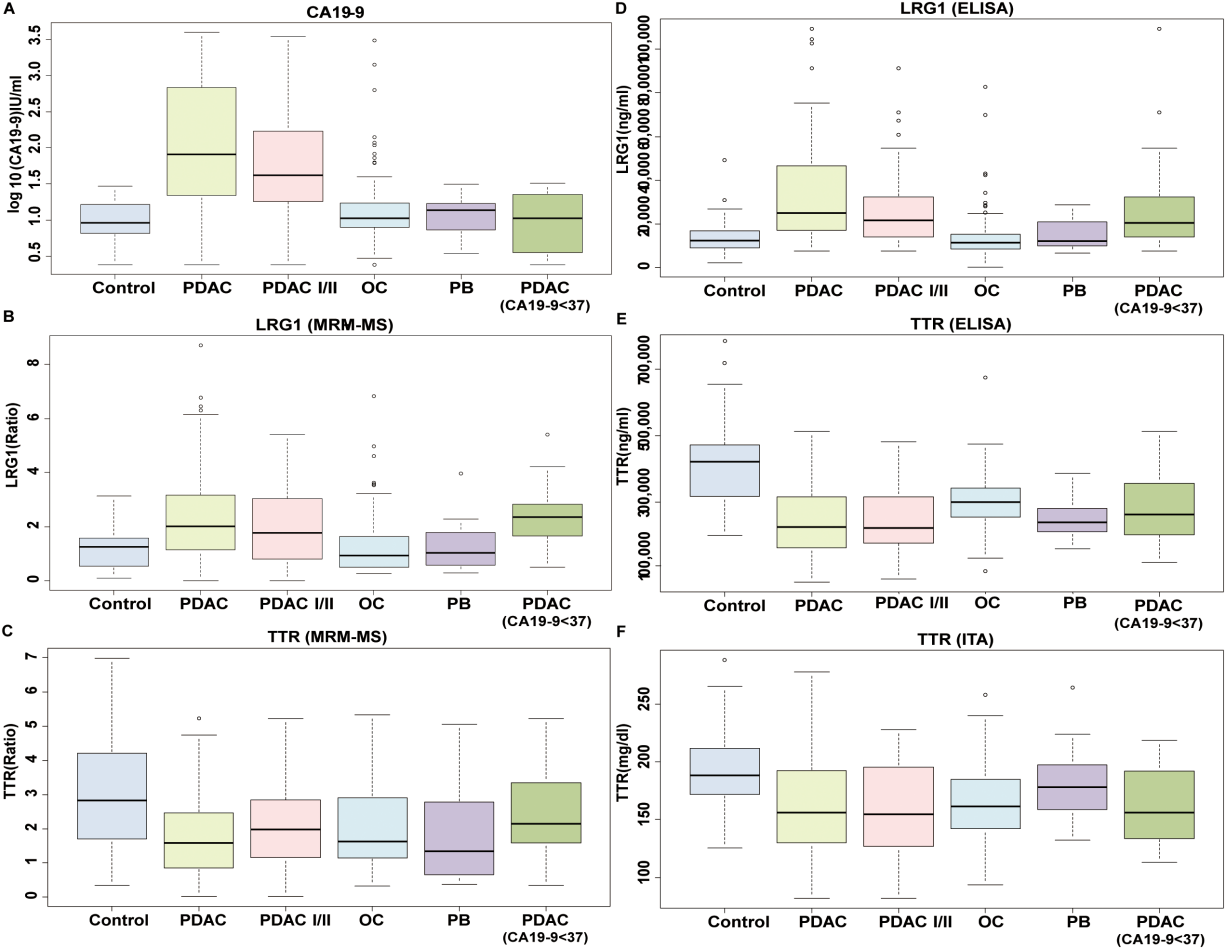
Box plots of expression of LRG1, TTR, and CA19-9 in all disease status The levels of **(A)** CA19-9, **(B)** LRG1, and **(C)** TTR were measured in control, all stages of PDAC, stage I/II of PDAC, other cancers, pancreatic benign disease, and all stages of PDAC with low levels of CA19-9. LRG1 and TTR were measured by MRM-MS and are shown as a ratio of light to heavy peptides. CA19-9 levels were measured by immunoassay and are given in log_10_ (U/mL). The levels of **(D)** LRG1, and **(E)** TTR were also evaluated by ELISA. The concentration of **(F)** TTR was also measured by immunoturbidimetric assay (ITA) due to its molecular characteristics. The ELISA results were shown in ng/ml, whereas immunoturbidimetric assay results were given in mg/dl. CA19-9 and LRG1 tended to increase in PDAC, whereas TTR was decreased, regardless of immunoassay type. Even when CA19-9 levels were lower than 37 U/mL in PDAC patients, LRG1 and TTR levels were distinctive.

**Figure 3:**
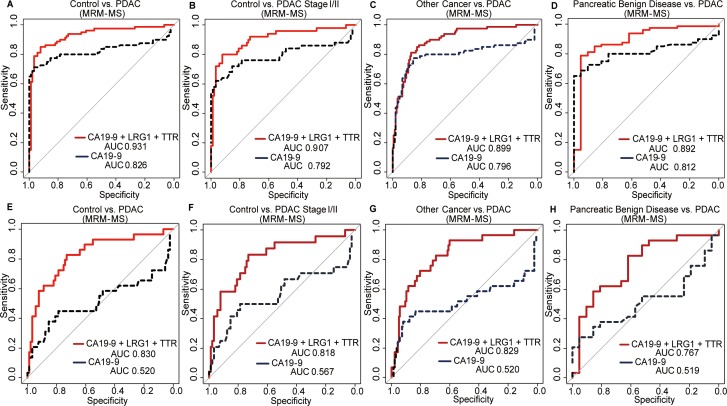
Receiver operating characteristic (ROC) curves for the triple-marker panel and CA19-9 in various settings The general performance was examined for **(A)** control vs. PDAC, and early detection was evaluated for **(B)** control vs. stage I/II PDAC. For selectivity, **(C)** other cancers vs. PDAC and **(D)** pancreatic benign disease vs. PDAC were analyzed. The ROC curve of CA19-9 and the panel as measured by MRM-MS was also generated for patients with < 37 U/mL CA19-9 for **(E)** control vs. all stages of PDAC, **(F)** control vs. stage I/II PDAC, **(G)** other cancers vs. all stages of PDAC, and **(H)** pancreatic benign disease vs. all stages of PDAC. CA19-9 had an AUC value of approximately 0.5 under all conditions; yet, the triple-marker panel had an AUC value of at least 0.767.

The final panel proteins (LRG1, TTR, and CA19-9) were tested on 1002 samples—composed of normal, benign, other cancers, and PDAC—which were divided into training (n=684) and test sets (n=318) at a 4:1 ratio (Supplementary Table 4). All AUC values were calculated by applying the classifier that was determined from the training set to the test sets. Two-class support vector machine (SVM) was performed to classify all group comparisons (Table 3). One group comprised PDAC patients, and the other groups had various compositions for the classification purposes. Box plots (Figure 2) and receiver operating characteristic curves (Figure 3) for the triple-marker panel and CA19-9 were generated. DeLong's method and McNemar test were applied to compare two classifiers of the single CA19-9 and triple-marker panel (CA19-9+LRG1+TTR). DeLong's method was used to determine whether two classifiers had the same AUC value. McNemar test was performed to show diagnostic homogeneity of the two classifiers. The sensitivity and specificity were obtained by applying the cut-off value from the training set when the specificity was set to 90.0%. The performance is summarized in Table 3.

The overall performance of the panel was examined for all PDAC samples and controls, and its ability with regard to early detection was evaluated in stage I/II PDAC compared with controls. In the first test set (Test 1, Supplementary Table 4) of 80 cases and 89 controls, the panel, as measured by MRM-MS, had an AUC value of 0.931 (sensitivity = 82.5%), which was 11% higher than that of CA19-9 alone (AUC = 0.826 when sensitivity = 72.5%) (Figure 3A). Similarly, by Immunoassay 2, the AUC value was 0.932 (Table 3). The levels of CA19-9 and LRG1 increased, and TTR declined in the case group (Figure 2A to 2F).

To distinguish surgically operable early-stage PC, 50 samples from only stages I and II were compared with 89 controls (Test 2, Supplementary Table 4). CA19-9 had an AUC value of 0.792 (sensitivity = 64.0%). By MRM-MS and Immunoassay 2, the AUC values were 0.907 and 0.914 with a sensitivity of 76.0% and 78.0%, respectively (Figure 3B, Table 3). The concentrations of CA19-9, LRG1, and TTR also changed in the early stages (Figure 2). These results indicate that the multimarker panel, rather than the individual markers, predicts the early stages of PDAC.

Although our panel had high sensitivity and specificity, its false positive detection of patients can lead to unnecessary examinations. Thus, we calculated the positive predictive value of the triple-marker panel, based on the actual prevalence of pancreatic cancer, which is 12.9 patients per 0.1 million people [35]. The positive predictive value (PPV) was calculated as follows:

PPV=(Sensitivity×P(D))/(Sensitivity×P(D)+(1-Specificity)(1-P(D)))

where P(D) is 12.9/100,000, or the prevalence of pancreatic cancer patients in Korea. In group comparisons except other cancer versus PDAC, our triple-marker panel had improved PPV values compared with CA19-9 (Supplementary Table 6). However, the overall PPV values were low due to the low prevalence of pancreatic cancer.

### Selectivity of triple-marker panel for PC

The specificity of the panel was measured in other cancers, such as breast (n=52), colon (n=45), and thyroid (n=52) (Test 3, Supplementary Table 4). Compared with CA19-9 (AUC=0.796), the panel discriminated PDAC from other cancers better, based on a 10% increase in AUC values. For 80 cases versus 149 other cancers, the AUC values by MRM-MS and Immunoassay 2 were 0.899 and 0.898, respectively (Figure 3C, Table 3). When the specificity was 83.9%, the sensitivity was 82.5%—10% higher than that of CA 19-9 (72.5%) (Table 3). CA19-9 and LRG1 levels rose, whereas TTR decreased, regardless of platform (Figure 2A to 2F).

To select PDAC from benign pancreatic growth, pancreatic cancer (n=80) was distinguished from benign pancreatic disease (n=21) (Test 4, Supplementary Table 4). CA19-9 and LRG1 levels were higher compared with TTR, which was decreased (Figure 2A to 2F). By MRM-MS, CA19-9 had an AUC value of 0.812 with a specificity of 81.0% and sensitivity of 72.5%, whereas the triple-marker panel improved its AUC value to 0.892 with a specificity of 85.7% and sensitivity of 82.5% (Figure 3D). Our panel had an AUC value of 0.895 (specificity=85.7%, sensitivity=82.5%) by Immunoassay 2 (Table 3). The results demonstrate that the triple-marker panel distinguishes PDAC from other cancers and benign diseases.

### Improved performance of panel in patients with normal CA19-9 levels

Our panel was then tested on subjects who were within the normal range of CA19-9 (< 37 U/mL), because PC patients, primarily in the early stages, could not be differentiated (Figure 3E to 3H, Test 5, Supplementary Table 4). In the low-CA19-9 groups, the AUC value of CA19-9 was approximately 0.500, which demonstrated a lack of discriminatory power against any controls. In contrast, by MRM-MS and Immunoassay 2, the triple-marker panel had an AUC value of 0.830 and 0.829, respectively (Figure 3E, Table 3). The sensitivity, when the specificity was adjusted to roughly 90.0%, was also improved by 28% (sensitivity=51.7%) in control versus PDAC compared with the sensitivity of CA19-9, which was 24.1% (Table 3).

The performance of the panel in PC patients with CA19-9 levels within the reference range was evaluated under various conditions: controls vs. stage I/II PDAC, pancreatic benign disease vs. PDAC, and other cancers vs. PDAC. Each protein was measured individually, wherein LRG1 and TTR levels rose and decreased, respectively, and CA19-9 level was unchanged (Figure 2A to 2F). The AUC value of CA19-9 in every test setting was approximately 0.500. Early detection of PDAC improved, based on the increase in AUC value to 0.818 by MRM-MS and immunoassay (Figure 3F and Table 3). PDAC could be differentiated from other cancers, based on an AUC value of 0.829 by MRM-MS (Figure 3G and 3E). Benign and malignant pancreatic disease could be distinguished by the triple-marker panel, with an AUC value of 0.767, compared to 0.519 for CA19-9 (Figure 3H and Table 3). The immunoassay data correlated with these results in all test settings (Table 3 and Figure 2D to 2F). Collectively, the multimarker panel complements the performance of the current marker, CA19-9, in the diagnosis of pancreatic cancer.

## DISCUSSION

In this large-scale, retrospective, and multi-center study, we developed a multimarker panel to diagnose the early stages of PDAC using conventional immunoassays and a high-throughput assay, followed by an advanced statistical machine-learning approach, to analyze the proteomic phenotype that is associated with genetic mutations or is functionally linked to pancreatic cancer. Plasma LRG1 and TTR levels, with CA19-9, had greater diagnostic value for PDAC than CA19-9 alone (at least a 10% increase in AUC value and sensitivity at 90% specificity for all cases). Regardless of the range of CA19-9 values, the multimarker panel assessed PDAC more accurately. In addition, as the disease lesion exacerbated, the levels of these markers rose or decreased, distinguishing early-stage cancer from benign diseases. These results imply that the triple-marker panel differentiates PC patients from inflammatory and other disease states in high-risk populations through regular health screens.

Biomarker discovery results in myriad candidate proteins that must be verified in various patient samples, but the verification of disease biomarkers delays their clinical translation. This study is significant, because we proceeded from the discovery to the validation of candidate markers in various platforms. Protein markers must be specifically overexpressed in certain cancers, generate stable and reproducible results, and be validated accurately in large cohort studies to be introduced to the clinic. If these requirements are met, protein markers have tremendous potential to become a routine clinical application, given that blood tests are less invasive, cost-effective, and require small amounts of plasma. Thus, our goal was to perform a rapid verification of potential proteins and validate them by traditional antibody-based assays. To this end, we introduced an alternative approach, MRM-MS–a continuous process from the discovery to the validation of potential biomarkers. The lengthy lists of all possible targets from extensive literature searches, public databases, and microarrays from tissues in PC, benign state, and normal patients enriched our study. The performance of our panel was evaluated in plasma samples with various disease statuses. The targets were validated in large-scale samples using several assays to confirm the results. A triple-marker panel (LRG1, TTR, and CA19-9) might lead to early diagnosis, reduce the costs of screening and treatment, and lengthen survival (disease-free interval). It would also improve the quality of life of PC patients, because fewer invasive procedures would be performed and ineffective treatments would be withdrawn.

The origin and function of LRG1 and TTR are unclear to date. However, there has been a report that TTR, which is synthesized by pancreatic islets, is involved in pancreatic β-cell death, and insulin release [36]. TTR is decreased in type 1 diabetes mellitus, yet is highly abundant in PC juice, because the pancreatic islet is destroyed, allowing proteins to leak into the pancreatic ductal system [37, 38]. This contradictory result might be attributed to differences in sample types. TTR is also synthesized in the endocrine pancreas, liver, and choroid plexus of the brain, and endocrine pancreatic tumors contain TTR mRNA, corresponding to our previous microarray result [32, 39]. In addition, PDAC patients often experience malnutrition, lowering the levels of TTR, which is involved in energy intake, acute/chronic disease states, nutritional status, and inflammatory processes [40]. As the level of TTR declines during the progression of PC, TTR might originate from somewhere other than the cancer. However, systemic changes in cancer patients can alter certain proteins and represent the early physiological changes of cancer. LRG1 mediates angiogenesis and TGF-β signaling [41]. LRG1 levels are elevated in the blood of patients with non-small-cell lung [42], ovarian [43], colorectal cancer [44], and gliomas [45] through TGF-β signaling, which promotes endothelial cell proliferation and angiogenesis [46]. Elevation of LRG1 in PC patients has been reported [47], but we improved the diagnostic performance of LRG1 by combining TTR and CA19-9. Subsequently, the triple-marker panel was tested in a larger cohort, including more cases that were in the resectable stages of pancreatic cancer and other cancer samples. Migration and invasion of hepatocellular carcinoma cells are suppressed by LRG1 [48]. A recent study reported that LRG1 is associated with endothelial dysfunction, arterial stiffness, and peripheral arterial disease in patients with type 2 diabetes [49]. The specific functions of each molecule in the panel must be determined, as should their molecular mechanisms and clinical significance. We expect that the small mass of an early cancer is less likely to change the molecular composition of peripheral blood dramatically. Rather, the systemic responses to abnormal changes in the organ might regulate the molecules that can be detected in the plasma by all 3 of the platforms that we used. Although there should be a deliberate consideration, our results suggest that LRG1 and TTR are highly relevant to PC.

Current studies on blood markers are examining the complexity of biological and individual variability. Moreover, PC is one of the most challenging cancers to evaluate, necessitating considerable research. The major limitation of this study was its retrospective sample collection, due to the retrospective exploratory nature of the translational research. Thus, further clinical validation is needed to determine the appropriateness of the panel in prospective screening tests and its practical feasibility. Focusing on patients with stage I pancreatic cancer is needed to form the optimal screening cohort. In this study, only 4 samples with stage I PC were included in the test set due to the low detection of stage I PC [50–52]. Thus, a comparison of control (normal control or benign pancreatic disease) and only stage I PC patients would not be statistically significant due to the low number of stage I samples. However, the main purpose of this study was to diagnose resectable stage I or II PC (n=50), which might improve overall survival through surgical resection and systemic therapy. The reliability of the kit should be tested in follow-up analyses, and its risk for generating false positives should be evaluated. Further, although this report is one of the largest validation studies on this subject, only one ethnic group was recruited, limiting its generalizability to all populations worldwide. However, all samples, except for the normal samples, were obtained from many centers, reducing institutional bias. We also tested the final panel in several independent cohorts. Trends of the marker levels in the same subject over time (from years before diagnosis of the cancer to the time of diagnosis) would be helpful in a future study.

This study is the first multicenter and large-scale corroboration of the clinical diagnostic value of LRG1 and TTR among many documented candidates. This paper thus provides reliable evidence of the relationship between LRG1 and TTR with the early stages of PDAC and the diagnostic performance of the panel in distinguishing PDAC from normal, benign disease states, and the patients with other cancers (colorectal, thyroid, and breast cancer, in particular). Our findings indicate that the multimarker panel can guide medical decisions with regard to the patients in their early stages or with low CA19-9 levels. Furthermore, determining the function of target proteins and peptides in a tumor microenvironment, including the pathways in which they are involved, can help identify potential targets for treatment and increase our understanding of cancerous environments.

## MATERIALS AND METHODS

### Experimental design

To develop a multimarker panel for the early detection of pancreatic cancer, we generated and tested a panel by extensive target selection and validation in a large retrospective cohort from several institutions.

Over 1000 samples were allocated to the discovery/verification (n=134) and validation sets (n=1008). A total of 684 plasma samples (316 PDAC, 88 PB, and 280 NL) constituted the training set. The verified proteins were rescreened in the test set of 318 plasma samples (80 PDAC, 21 PB, 68 NL, and 149 OC). Samples from benign diseases, such as pancreatitis, were collected to distinguish cancer from benign states. Samples of other cancers (thyroid, breast, and colon) were included to assess cancer specificity. Because most pancreatic cancer patients were diagnosed in the metastatic or unresectable stage, we focused on resectable stage or early-stage disease (stage I and II), which might be treated successfully with surgical resection, followed by systemic therapy [50–52]. All samples were considered for biological factors, such as age, gender, BMI, and smoking history. In each sample preparation, blinding and blocked randomization were performed to negate any subjective bias of the sample groups.

All targets were measured by MRM-MS, and differentially abundant proteins, as determined by SVM learning, were assembled into a multimarker panel. The verification of single markers was performed in triplicate, whereas the validation was performed once in a large clinical sample to strengthen the reliability. Two models (CA19-9 and multimarker panel) were trained in the training set, fixed, and then applied to the independent test set to obtain the AUC values [53]. The final targets (LRG1 and TTR) were also measured by immunoassay. The performance of the panel was determined by its sensitivity and specificity.

### Clinical plasma sample preparation for MRM-MS analysis

High abundant plasma proteins were immune-depleted on a multiple affinity removal system (MARS) column, concentrated, digested by trypsin, and desalted as described previously [13]. The prepared samples were frozen, lyophilized on speed vacuum centrifuges, and stored at -80°C until analysis. The samples were resolubilized in mobile phase A to 2 μg/μL and spiked with stable isotope-labeled standard (SIS) peptide, as needed. More details on the protocol are provided in Supplementary Materials.

### Verification and validation of markers by quantitative MRM-MS assay

Individual samples were analyzed by LC-MS/MS on a 6490 triple quadrupole (QQQ) mass spectrometer (Agilent Technologies, Santa Clara, CA) that was equipped with ESI (iFunnel Technology source) and a capillary flow LC for the verification of prescreened candidate markers. Three transitions/peptides and a transition that showed the highest peak intensity were used for quantitation. Buffer A (0.1% formic acid/distilled water) and buffer B (0.1% formic acid/acetonitrile) flowed through the C18 column (150 mm x 0.5 mm i.d., Agilent Zorbax SB-C18, 3.5-μm particle size) at 20 μL/min. The peptides were eluted on a linear gradient of mobile phase B from 3% to 35% for 50 min. The concentration was increased to 80% for 10 min and was reduced again to 5% for 10 min to equilibrate the column for the next run. The total LC run time was 70 min. The ion spray capillary voltage was 2500 V, and the nozzle voltage was 2000 V. The drying gas temperature was 250°C with a flow rate of 15 L/min. The sheath gas temperature was 350°C with flow rate of 12 L/min. The nebulizer gas was set to 30 psi, the fragmentor voltage was 380 V, and the cell accelerator voltage was 5 V. The delta EMV was set to 200 V. Quadrupoles 1 and 3 were maintained at unit (0.7 FWHM) resolution. Peptide RT and optimized collision energy values were supplied to MassHunter (vB06.01, Agilent Technologies) to establish a dynamic MRM-MS scheduling method, based on input parameters of 1500 ± 500-ms cycle times and 4-min retention time windows. Dwell times varied, depending on the number of concurrent transitions; in all cases, they were at least 5 ms. Min/max dwell times were established by the software, and the data were analyzed using Skyline.

### Validation by immunoassay

LRG1 was measured only by ELISA, and TTR was measured by ITA (Immunoassay 1) and ELISA (Immunoassay 2) due to its molecular characteristics and the lack of a commercial kit and references. Two targets, LRG1 and TTR, were tested using a commercial hLRG1 and prealbumin ELISA kit (IBL, Hamburg, DE, Germany and AssayPro, Saint Charles, MO, USA). All tests were performed according to the manufacturer's recommendations. The concentration was obtained by 4-parameter logistic curve-fit, multiplied by the dilution factors. The level of TTR was also measured using the COBAS© INTEGRA 800 Prealbumin kit (Roche Diagnostics, Basel, Switzerland).

### Statistical analysis

MRM-MS peak integration was performed manually in Skyline. The peak area was normalized to that of beta-galactosidase (external standard) or the SIS in the same run. Statistical analyses and graphical works were conducted using SPSS 16.0 for Windows (SPSS Inc. Chicago, IL, USA), MedCalc 10.4.7.0. (MedCalc Software, Mariakerke, Belgium, version 10.0.1.0), R ver. 3.2.1 (R Foundation for Statistical Computing, Vienna, Austria), and GraphPad Prism 5 (San Diego, CA).

Differential expression between independent groups was analyzed by SVM [54]. The sample groups were divided into training and test sets. The function of a diagnosis of pancreatic cancer was as follows:

$$f(x)=\mathrm{sgn}(\sum_{i=1}^{n}\alpha_{i}y_{i}<x,x_{i}>+b)$$

where *x* is the newly measured level of CA19-9, LRG1, and TTR; α_i_ is the multiplier of Lagrange; *y_i_* is the identifier of normal or PC patients; *x*_*i*_ is the standard measured value; and *b* is the correction value. The function was applied to the test set, and PC was identified when f(x) was 1, whereas the normal population was classified when f(x) was −1. Specificity and sensitivity were assessed using ROC curves, represented by corresponding AUC values with 95% CI. DeLong's test was used to evaluate AUC values, and McNemar's test was used to analyze the diagnostic performance of the combined panel when the specificity was 90%. Two-sided p-values < 0.05 were considered to be significant.

## SUPPLEMENTARY MATERIALS FIGURES AND TABLES







## Abbreviations

AMC: Asan Medical Center
AUC: area under the curve
AuDIT: automated detection of inaccurate and imprecise
BLAST: basic local alignment search tool
CT: computed tomography
CV: Coefficient of variance
ELISA: enzyme-linked immunosorbent assay
ESI: electrospray ionization
EUS: endoscopic ultrasound
FDA: US Food and Drug Administration
ITA: immunoturbidimetric assay
LC: liquid chromatography
LRG1: leucine-rich alpha-2 glycoprotein
MARS: multiple affinity removal system
MRM-MS: multiple reaction monitoring-mass spectrometry
NCC: National Cancer Center
NIST: National Institute of Standards and Technology
NL: normal control
OC: other cancer
PB: pancreatic benign
PC: pancreatic cancer
PDAC: pancreatic ductal adenocarcinoma
QqQ: triple quadrupole
ROC: receiver operating characteristic
SIS: stable isotope-labeled synthetic
SMC: Samsung Medical Center
SNUH: Seoul National University Hospital or Seoul National University Hospital Healthcare System Gangnam Center
SRM: selected reaction monitoring
SVM: support vector machine
TTR: transthyretin
YSH: Yonsei Severance Hospital.
