# Correction: Diagnostic performance enhancement of pancreatic cancer using proteomic multimarker panel

**DOI:** 10.18632/oncotarget.24175

**Published:** 2018-01-12

**Authors:** Jiyoung Park, Yonghwan Choi, Junghyun Namkung, Sung Gon Yi, Hyunsoo Kim, Jiyoung Yu, Yongkang Kim, Min-Seok Kwon, Wooil Kwon, Do-Youn Oh, Sun-Whe Kim, Seung-Yong Jeong, Wonshik Han, Kyu Eun Lee, Jin Seok Heo, Joon Oh Park, Joo Kyung Park, Song Cheol Kim, Chang Moo Kang, Woo Jin Lee, Seungyeoun Lee, Sangjo Han, Taesung Park, Jin-Young Jang, Youngsoo Kim

**Affiliations:** ^1^ Department of Biomedical Sciences, Seoul National University College of Medicine, Seoul, Korea; ^2^ Department of Biomedical Engineering, Seoul National University College of Medicine, Seoul, Korea; ^3^ Immunodiagnostics R&D Team, IVD Business Unit 5, SK Telecom, Seoul, Korea; ^4^ Department of Statistics, Seoul National University, Seoul, Korea; ^5^ Department of Surgery and Cancer Research Institute, Seoul National University College of Medicine, Seoul, Korea; ^6^ Department of Internal Medicine and Cancer Research Institute, Seoul National University Hospital, Seoul, Korea; ^7^ Department of Surgery, Samsung Medical Center, Sungkyunkwan University School of Medicine, Seoul, Korea; ^8^ Internal Medicine, Samsung Medical Center, Sungkyunkwan University School of Medicine, Seoul, Korea; ^9^ Department of Internal Medicine, Seoul National University Hospital Healthcare System Gangnam Center, Seoul, Korea; ^10^ Department of Surgery, University of Ulsan College of Medicine and Asan Medical Center, Seoul, Korea; ^11^ Department of Surgery, Severance Hospital, Yonsei University College of Medicine, Seoul, Korea; ^12^ Center for Liver Cancer, National Cancer Center, Seoul, Korea; ^13^ Department of Mathematics and Statistics, Sejong University, Seoul, Korea; ^14^ School of Interdisciplinary Bioscience and Bioengineering, Pohang University of Science and Technology (POSTECH), Pohang, Korea

This article has been corrected: Yonghwan Choi is now listed in the 3^rd^ and 14^th^ affiliations and has been added to the equal contribution note.

Original article: Oncotarget. 2017; 8:93117-93130. https://doi.org/10.18632/oncotarget.21861

