# Supplementary material for: Diagnostic performance enhancement of pancreatic cancer using proteomic multimarker panel
Source: Oncotarget. 2017 Oct 16;8(54):93117–30. doi: 10.18632/oncotarget.21861 (PMC5696248; doi:10.18632/oncotarget.21861)
Supplement: Supplementary file 1 [file oncotarget-08-93117-s001.pdf]

Diagnostic performance enhancement of pancreatic cancer using proteomic multimarker panel

SUPPLEMENTARY MATERIALS

Collection of study samples

This retrospective cohort study was based on 1008 participants who were recruited from 5 major medical centers: National Cancer Center (NCC), Seoul National University Hospital or Seoul National University Hospital Healthcare System Gangnam Center (SNUH), Samsung Medical Center (SMC), Asan Medical Center (AMC), and Yonsei Severance Hospital (YSH). All plasma samples were collected in EDTA-coated tubes, labeled by subject identification number, aliquoted, and frozen until further study. Hemolytic samples were excluded. Pancreatic cancer (PC) samples were obtained from patients who underwent surgery or chemoradiotherapy for PC (Table 1).

For the initial discovery/verification study, 134 plasma samples from SNUH between January 2011 and December 2013 were used. Of the 134 samples, 50 were drawn from PDAC patients who underwent surgery, 34 patients had pancreatic benign diseases (PB), and the remaining 50 controls were from 25 patients with gallbladder stones/cholecystitis without severe inflammation and 25 healthy people who underwent a general medical examination. Patients with other malignant diseases or a history of malignancy were excluded. The validation set comprised 1008 samples, including those from the verification study. The subjects were all matched for gender, age, and other clinical factors, such as smoking history, to avoid bias.

A set of 1002 of the 1008 samples, due to lack of sample volume, was used to evaluate the panel (Supplementary Table 5). Of 396 cases, 248 samples were classified to be resectable (stage I, II), and 148 samples were unresectable (stage III, IV). Also, 348 normal, 109 benign pancreatic diseases, and 149 other cancer samples (thyroid, colorectal, and breast cancer) were set as controls. All samples were divided into a training and test set. Of 684 training sets, there were 316 case samples, of which 198 were resectable and 118 were unresectable. A total of 368 controls, consisting of 280 normal and 88 benign pancreatic samples (pancreatitis and other benign diseases, Table 1) were used. The test set for general performance consisted of 80 cases (50 resectable and 30 unresectable) and 89 controls (68 normal and 21 benign pancreatic disease). Tumor stages were classified per the 7th edition of AJCC [1]. For selectivity analysis of tumor types, breast (n=52), thyroid (n=52), and colorectal cancer (n=45) samples were included. Normal samples were obtained from individuals who had regular health checkups at SNUH and had no malignancies or other serious health conditions.

Sample size calculation

From previous plasma biomarker discovery studies and based on the recently FDA-approved marker CA19-9 for PC, the number of samples was ensured to obtain an AUC value > 0.780 with over 95% probability when the panel was modeled using markers with AUC values in the range of 0.800 and 0.950 [2]. The maximum standard error of the calculated AUC was given, and the sample size was examined by the desired minimum AUC. When the standard error was determined to be 0.015 and the ratio of cases to controls was set to 1:1, the total number of required samples was 228 to 946, whereas 200 to 904 samples were needed when the proportion of cases to controls was 3:2. Because the case:control ratio did not affect the sample size dramatically, we tried to attain sample sets that were adjusted to 1:1.

Selection of candidate biomarkers by database and literature searches

We selected PC-related targets from 17 publications that were related to PC and proteomics; Oncomine [3], an integrated data-mining platform of cancer microarrays; MetaCore [4]; and Pathway Studio [5]—the procedures are summarized in Supplementary Figure 1. The filter parameters in Oncomine were as follows: analysis type was ‘cancer vs. normal analysis,’ and cancer type was ‘pancreatic cancer.’ The keyword ‘pancreatic cancer’ was used to identify targets from MetaCore and Pathway Studio. Cell localization was set to membrane or secreted proteins to focus on plasma proteins.

We built a cancer-protein relation network with the target proteins. Protein-protein interactions, direct regulation, microRNA effects, and transcription factor effects were used as the relationships between entities. To select PC-specific targets, 10 common cancers, such as gastric, breast, and colorectal, were connected. The shortest path from PC to each node of proteins was selected. Any paths that were related to other cancers were excluded; thus, only paths for PC were considered (Supplementary Figure 1A). Further, cancer-specific mutated proteins from databases or previous MS experiments were included. All

targets from various sources were merged and organized by matching accession numbers for the same proteins and removing overlapping genes. Organized targets were filtered using the plasma proteome database to remove undetectable proteins in plasma. Proteomics experiments that were related to PC were all performed in serum or plasma samples. Candidates that appeared in multiple databases or had documented evidence in the literature were chosen.

From 17 publications that were related to PC and proteomics, 819 proteins were selected as candidates. Eight datasets were obtained from ONCOMINE. A total of 8145 genes were screened. Further, 18 cancer-specific mutated proteins from databases or previous MS experiments were included, and 753 proteins were selected from MetaCore and Pathway Studio. Proteins from the publications and the Plasma Proteome Database (PPD) were merged, and plasma proteins were selected. Forty-four candidates were identified in all 4 resources, and 251 genes were included in 3 datasets. The 1225 targets in the 2 datasets were filtered by protein and mRNA expression level, p-value, and number of reported references. Thirty-nine targets from the proteomics articles and Oncomine database with more than 3 references were selected, because they showed differential expression of protein and mRNA. From the DEG papers and proteomics journals, 21 targets had differentially expressed protein and mRNA levels with p-value < 0.01. According to the commercial database, 36 proteins were differentially expressed in proteomics journals and Metacore or pathway studio analysis with more than 2 references. Of the targets with more than 2 references and differential expression in PC, 25 were present in Metacore or Pathway studio and the Oncomine database. Three targets appeared in the database and DEG analysis with p-value < 0.01. Of those with more than 3 references in the Oncomine and DEG analysis with p-value < 0.00001, 33 targets showed differential expression of mRNA. As a result, 157 genes were selected. Fifty-six genes that only appeared in 1 of the resources were included in the list of final targets, because they had more than 3 references in all resources.

Consequently, a total of 508 genes were selected for further analysis (Supplementary Figure 1B). In addition, 18 mutated proteins (8 proteins) that are mutated in PC—ADH1B_H48R, (wild-type ADH1B could not be detected due to its short length), AGER, AGER G28S, CFTR, CFTR G458V, SERPINB5, SERPINB5 I319V, SOD2, SOD2 V16A, SPINK1, SPINK1 Y43S, GNAS, GNAS R201C, GNAS R201H, KRAS, KRAS G12V, KRAS G12D, and KRAS G12R—were also considered to be targets. The targets of CFTR, SERPINB5, SOD2, and SPINK1 were initially included in the 508 candidates from the data-mining, so 14 proteins were labeled as known mutated proteins in Supplementary Table 2.

Selection of candidate biomarkers by microarray analysis

The microarray data were from a previous study; thus, all details on the microarray-related methods have been described [6]. Frozen pancreatic ductal adenocarcinoma (PDAC) (n=104), IPMN (n=50) and normal (n=17) tissues were used for an Affymetrix (Santa Clara, CA, USA) HuGene 1.0 ST (33,297 probes) mRNA array. All specimens were collected between 2008 and 2012 from Seoul National University Hospital. Clinicopathologic characteristics were defined per the 7th edition of AJCC. No patients had received any type of cancer therapy before the procedure. The experiments were approved by Seoul National University Hospital. The differential mRNA expression data were analyzed by support vector machine (SVM) classification and leave-one-out cross validation (LOOCV) [6]. All mRNAs that changed by more than 2-fold in normal and cancer tissues or had an absolute significance analysis microarray (SAM) score > 6 were included. Targets with q-value < 1 in benign pancreatic disease samples were also analyzed. mRNAs with similar expression levels in any of 2 categories (cancer, normal, and benign pancreatic disease) were eliminated due to lack of discriminatory power. For example, candidates that were differentially expressed in normal and benign pancreatic disease but not between normal and malignancy were excluded. The proteins that corresponded to the target mRNAs above were included in our candidate biomarker list. All procedures are summarized in Supplementary Figure 1C.

Plasma sample preparation

The 6 highly abundant plasma proteins (albumin, transferrin, IgG, IgA, haptoglobin, and α1-antitrypsin) were removed by high-performance liquid chromatography (HPLC; Shimadzu Co, Kyoto, Japan) with a multiple affinity removal system (MARS) column (Hu-6HC; 4.6 × 100 mm; Agilent Technologies, Santa Clara, CA). Each sample was diluted by a factor of 5 with MARS buffer A (Agilent, CA, USA) and passed through 0.22-μm Spin-X filters (Corning Costar, NY, USA) by centrifugation at 12,000 × *g*. To isolate low-abundant proteins, 200 μl of sample was injected at 0.25 mL/min. The cycle of loading, wash, elution, neutralization, and re-equilibration lasted 28 min. The bound and unbound portions were monitored on a chromatogram at 280 nm. Flow-through fractions that contained low-abundant proteins were pooled and stored at -80°C until use.

Depleted samples were concentrated by centrifugal filtration using a molecular weight cutoff (MWCO) of 3000 Da (Amicon Ultra-4 3K, Millipore, MA, USA). Samples were centrifuged for more than 6 hours at 4°C until the final volume of 50 to 100 µL. Concentrations of prepared samples were measured by bicinchoninic acid (BCA) assay. The concentrated sample (100 µg) was denatured and reduced with 6 M urea, 100 mM Tris, pH 8.0, and 20 mM dithiothreitol (DTT, Merck, Darmstadt, Germany) at 37°C for 60 min and alkylated with 50 mM iodoacetamide (IAA, Sigma, St. Louis, MO, USA) at room temperature (RT) in the dark for 30 min. To avoid trypsin compatibility with urea, the alkylated sample was diluted tenfold with 100 mM Tris, pH 8.0 prior to incubation with a stock solution of trypsin (Sequencing-grade modified, Promega, WI, USA) at an enzyme-to-substrate ratio of 1:50. After 16 hours of incubation at 37°C, 100% formic acid (FA) was added to a final concentration of 2% to quench the enzymatic reaction.

Then, digested samples were desalted using Oasis® HLB 1 cc (30 mg) extraction cartridges (Waters, MA, USA). The Oasis cartridge was washed with 1 mL 100% MeOH and 3 mL 100% acetonitrile (ACN) in 0.1% FA and equilibrated with 5 mL of 0.1% FA sequentially. The entire volume of the digested serum was then loaded onto the cartridge, and the cartridge was washed with 5 mL 0.1% FA. The sample was eluted with 0.5 mL 40% ACN in 0.1% FA and 0.8 mL 60% ACN in 0.1% FA. The eluted samples were frozen and lyophilized on a speed vacuum centrifuge and stored at -80°C until analysis. The sample was resolubilized in mobile phase A to 2 µg/µL and spiked with stable isotope-labeled standard (SIS) peptide, as needed.

Preliminary screen of target candidates by MRM-MS assay

A pooled plasma sample was prepared by mixing equal amounts of 6 plasma samples each from the normal control and PC groups. First, 50 fmol of the beta-galactosidase peptide GDFQFNISR($^{13}C^{15}N$) was spiked into every sample as an external peptide standard. Detectability was checked by a nano LC that was connected to a hybrid triple quadrupole/ion trap mass spectrometer (4000 QTRAP, AB SCIEX, Foster City, CA), equipped with a nanoelectrospray interface. Buffer A (97% DW, 3% Acetonitrile (ACN), 0.1% FA) and buffer B (3% DW, 97% ACN, 0.1% FA) flowed through the C18 column (3-µm bead size, 100 µm ID, 150 mm, 100-Å pore size, Michrom Bioresources, CA, USA) at a flow rate of 300 nL/min. The LC gradient started at 97% buffer A and 3% buffer B, followed by 3% to 35% buffer B for 50 min, 35% to 80% buffer B for 10 min, and 5% buffer B for 10 min. Typical instrument settings were as follows: ion spray (IS) voltage of 2.1 kV, an interface heater temperature of 180°C, a GS1 (nebulizer gas) setting of 12, and curtain gas set to 15 L/min. MS parameters for declustering potential (DP) and collision energy (CE) were determined by linear regression of previously optimized values in Skyline. MRM-MS experiments were performed with a scan time of 1.5 seconds and a scan width of 0.002 m/z, using a unit resolution of 0.7 Da (FWHM) for quadrupole part 1 (Q1) and quadrupole part 3 (Q3). The total LC run time was 60 min. The MRM-MS method was generated by 200 transitions per run, and a total of 114 runs were performed to quantify 907 proteins.

All raw files were processed using Skyline (McCoss Lab, University of Washington, USA) for the generation of extracted ion chromatograms and peak integration. We initially monitored 10 transitions per peptide to ensure specificity. Following peak detection and integration, peptides were considered to be “detectable” for each subject if: (i) the peptide transitions had a signal-to-noise ratio of ≥ 3, (ii) at least 4 light MRM-MS transitions were observed, and [7] the peptide had the same elution profile and ratios as the spectral library (dot product > 0.6). We configured these peptide candidates for multiplex MRM-MS assay, with SIS peptides used as internal standards. To examine the interference of endogenous target peptides in the subjects, 217 crude unpurified peptides (176 proteins) standards that corresponded to the detected endogenous peptides were synthesized with heavy isotopic lysine ($^{13}C6^{15}N2$, 8 Da mas shift) or arginine ($^{13}C6^{15}N4$, 10 Da mass shift) at the C-termini (SIS peptides). All SIS peptides were synthesized by JPT (Berlin, Germany) as crude unpurified SIS peptides.

Allocation of samples to all processing steps

A total of 1008 samples were divided into 4 batches for MRM-MS analysis. In each batch, 250 samples were organized at similar proportions of the disease states. The last batch had 8 additional samples. For quality assessment, pooled samples were allocated randomly at any position within the batch as a positive control. Depletion and MRM-MS analysis were analyzed within the same batch. Denaturation, digestion, and desalting were performed in parallel. Subjective bias of sample groups was negated by blocked randomization and blinding. Equal numbers of cases and controls were placed as much as possible in each randomly sized block.

Selection of observable peptides for MRM-MS analysis

We selected 1–20 peptides for each PC-related protein, based on the sequence of proteins in the Uniprot Knowledgebase (UniprotKB, http://www.uniprot.org/)

database. Transitions were generated in Skyline (McCoss Lab, University of Washington, USA). The peptide/transition selection criteria were as follows: (i) length 7–24 amino acid residues; (ii) signal peptides in the N-terminus were excluded due to hydrophobicity; [7] peptides that contained methionine (Met), histidine (His), N-linkage sites (NXT/NXS), and proline next to arginine or lysine (RP/KP) were not considered due to modification, charge alteration, and digestion problems; (iv) fragment y ion was selected for 2^+ charge state; and (v) the MS spectrum range was 300~1400m/z. Q1 transitions were derived by peptides that satisfied the conditions above. A maximum of 10 Q3 transitions from the fragmented ions were used.

All peptides in this study were evaluated using Protein Basic Local Alignment Search Tool (BLAST, http://blast.ncbi.nlm.nih.gov/Blast.cgi) to ensure uniqueness of the target proteins at the proteomic level. The peptides shared no similarity to other proteins that were likely to be found in human plasma. Using SRM collider [8], unique peptide transitions were determined by comparing target transitions to all other transitions of a known proteome. Interferences were removed to reduce the number of false positive results. The background genome was "human peptideatlas." Retention time was predicted by setting the SSRcalc window to 10 arbitrary units. The mass windows for Q1 and Q3 were 0.7 and 1.0, respectively. The low mass threshold for transitions was 300 m/z, and the high mass threshold was 1500 m/z. The background ion series was set to b and y ions. Transitions that showed any interference were removed.

Then, unique peptides were computed, resulting in a total of 225 proteins. Target proteins were narrowed down by MRM-MS assay and verified in 134 plasma samples (50 pancreatic cancers, 34 pancreatic benign diseases, and 50 normal controls). To develop the MRM-MS assays, 217 SIS peptides were synthesized. Using the SIS peptides, relative quantitation of target peptides was performed in 134 individual samples.

Verification and validation of markers by quantitative MRM-MS assay

Individual samples were analyzed by LC-MS/MS on a 6490 triple quadrupole (QQQ) mass spectrometer (Agilent Technologies, Santa Clara, CA) that was equipped with ESI (iFunnel Technology source) and a capillary flow LC for the verification of prescreened candidate markers. Three transitions/peptides and a transition that showed the highest peak intensity were used for quantitation. Buffer A (0.1% formic acid/distilled water) and buffer B (0.1% formic acid/acetonitrile) flowed through the C18 column (150 mm x 0.5 mm i.d., Agilent Zorbax SB-C18, 3.5-µm particle size) at 20 µL/min. The peptides were eluted on a linear gradient of mobile phase B from 3% to 35% for 50 min. The concentration was increased to 80% for 10 min and was reduced again to 5% for 10 min to equilibrate the column for the next run. The total LC run time was 70 min. The ion spray capillary voltage was 2500 V, and the nozzle voltage was 2000 V. The drying gas temperature was 250°C with a flow rate of 15 L/min. The sheath gas temperature was 350°C with flow rate of 12 L/min. The nebulizer gas was set to 30 psi, the fragmentor voltage was 380 V, and the cell accelerator voltage was 5 V. The delta EMV was set to 200 V. Quadrupoles 1 and 3 were maintained at unit (0.7 FWHM) resolution. Peptide RT and optimized collision energy values were supplied to MassHunter (vB06.01, Agilent Technologies) to establish a dynamic MRM-MS scheduling method, based on input parameters of 1500 ± 500-ms cycle times and 4-min retention time windows. Dwell times varied, depending on the number of concurrent transitions; in all cases, they were at least 5 ms. Min/max dwell times were established by the software, and the data were analyzed using Skyline.

Reverse response curves and performance of MRM-MS

Reverse response curves were generated using pooled serum samples with 2 µg/µL of a mixture of SIS peptides to determine the linearity. The solution was diluted sequentially (1:2) with pooled serum (0 fmol of SIS peptides) to draw a calibration curve using 11 points (1250, 625.0, 312.5, 156.3, 78.1, 39.1, 19.5, 9.77, 4.88, 2.44, and 1.22 fmols per injection). Each point in the response curves reflected MRM-MS analyses of 3 to 4 transitions per peptide with 3 technical replicates. Transitions with the highest signals and lowest backgrounds were used to quantify the amounts of the corresponding proteins. The peak area ratio was fit by linear regression using the "1/y" (y = peak area ratio) size weighting option. We determined the lower limit of quantitation (LLOQ) as the minimum quantitation level with linearity $R^2 > 0.998$ and $CV < 20\%$. Each assay step was performed in triplicate (Supplementary Figure 3).

Validation by immunoassay

Plasma levels of 3 proteins were measured by immunoassays in all 1008 samples, except for 6 samples that did not have sufficient volume for analysis. Two targets, LRG1 and TTR, were tested using a commercial hLRG1 and prealbumin ELISA kit (IBL, Hamburg, DE, Germany and AssayPro, Saint Charles, MO, USA). All tests were performed according to the manufacturer's recommendations. The ELISA kit and samples were placed at room temperature before the assay. LRG1 and TTR were diluted by 1000- and 80,000-fold, respectively, using the designated solutions. The standard, control,

and samples (50 μL) were loaded onto each well. The standard and control were duplicated. The wells were covered with a sealer and incubated at room temperature for 2 hours. Then, the solution was discarded, and distilled water was added to wash the plate. After repeating the washing step 4 times, 200 μL of conjugate was added and incubated for 2 hours at room temperature. The wells were washed 4 times, and 200 μL of substrate solution was added and incubated at room temperature for 30 min. The reaction was ceased with 50 μL of stop solution. The optical density was measured at 540 nm or 570 nm. The concentration was obtained by 4-parameter logistic curve-fit, multiplied by the dilution factors. The level of TTR was also measured using the COBAS© INTEGRA 800 Prealbumin kit (Roche Diagnostics, Basel, Switzerland).

SUPPLEMENTARY REFERENCES

1. McIntyre CA, Winter JM. Diagnostic evaluation and staging of pancreatic ductal adenocarcinoma. Semin Oncol. 2015; 42: 19-27. http://dx.doi.org/10.1053/j.seminoncol.2014.12.003.
2. Koopmann J, Rosenzweig CN, Zhang Z, Canto MI, Brown DA, Hunter M, Yeo C, Chan DW, Breit SN, Goggins M. Serum markers in patients with resectable pancreatic adenocarcinoma: macrophage inhibitory cytokine 1 versus CA19-9. Clin Cancer Res. 2006; 12: 442-6. https://doi.org/10.1158/1078-0432.CCR-05-0564.
3. Rhodes DR, Yu J, Shanker K, Deshpande N, Varambally R, Ghosh D, Barrette T, Pandey A, Chinnaiyan AM. ONCOMINE: a cancer microarray database and integrated data-mining platform. Neoplasia. 2004; 6: 1-6.
4. Schuierer S, Tranchevent LC, Dengler U, Moreau Y. Large-scale benchmark of Endeavour using MetaCore maps. Bioinformatics. 2010; 26: 1922-3. https://doi.org/10.1093/bioinformatics/btq307.
5. Nikitin A, Egorov S, Daraselia N, Mazo I. Pathway studio—the analysis and navigation of molecular networks. Bioinformatics. 2003; 19: 2155-7. https://doi.org/10.1093/bioinformatics/btg290.
6. Kwon MS, Kim Y, Lee S, Namkung J, Yun T, Yi SG, Han S, Kang M, Kim SW, Jang JY, Park T. Integrative analysis of multi-omics data for identifying multi-markers for diagnosing pancreatic cancer. BMC Genomics. 2015; 16: S4. https://doi.org/10.1186/1471-2164-16-S9-S4.
7. Petricoin Iii EF, Ardekani AM, Hitt BA, Levine PJ, Fusaro VA, Steinberg SM, Mills GB, Simone C, Fishman DA, Kohn EC, Liotta LA. Use of proteomic patterns in serum to identify ovarian cancer. Lancet. 2002; 359: 572-7. http://dx.doi.org/10.1016/S0140-6736(02)07746-2.
8. Röst H, Malmström L, Aebersold R. A Computational tool to detect and avoid redundancy in selected reaction monitoring. Mol Cell Proteomics. 2012; 11: 540-9. https://doi.org/10.1074/mcp.M111.013045.

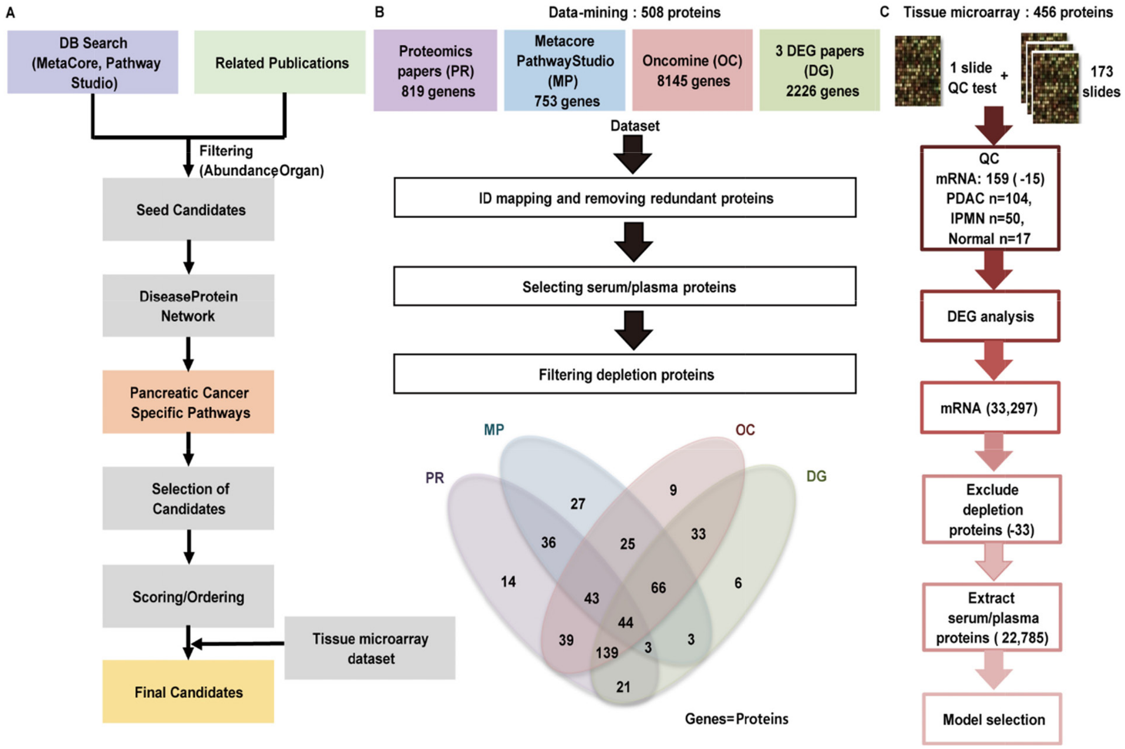

Supplementary Figure 1: Plasma proteome target selection. From pancreatic cancer- or proteomics-related papers, databases, and mRNA expression results, proteins with more than 3 references and 1 or 2 references that satisfied the filter conditions were selected. A total of 508 proteins were chosen from the data mining step of pancreatic cancer-related journals and public databases, such as MetaCore and PathwayStudio. All proteins from each source were filtered by removing redundant proteins and selecting plasma proteins. The remaining 456 proteins were selected by microarray and DEG (differentially expressed genes) analysis.

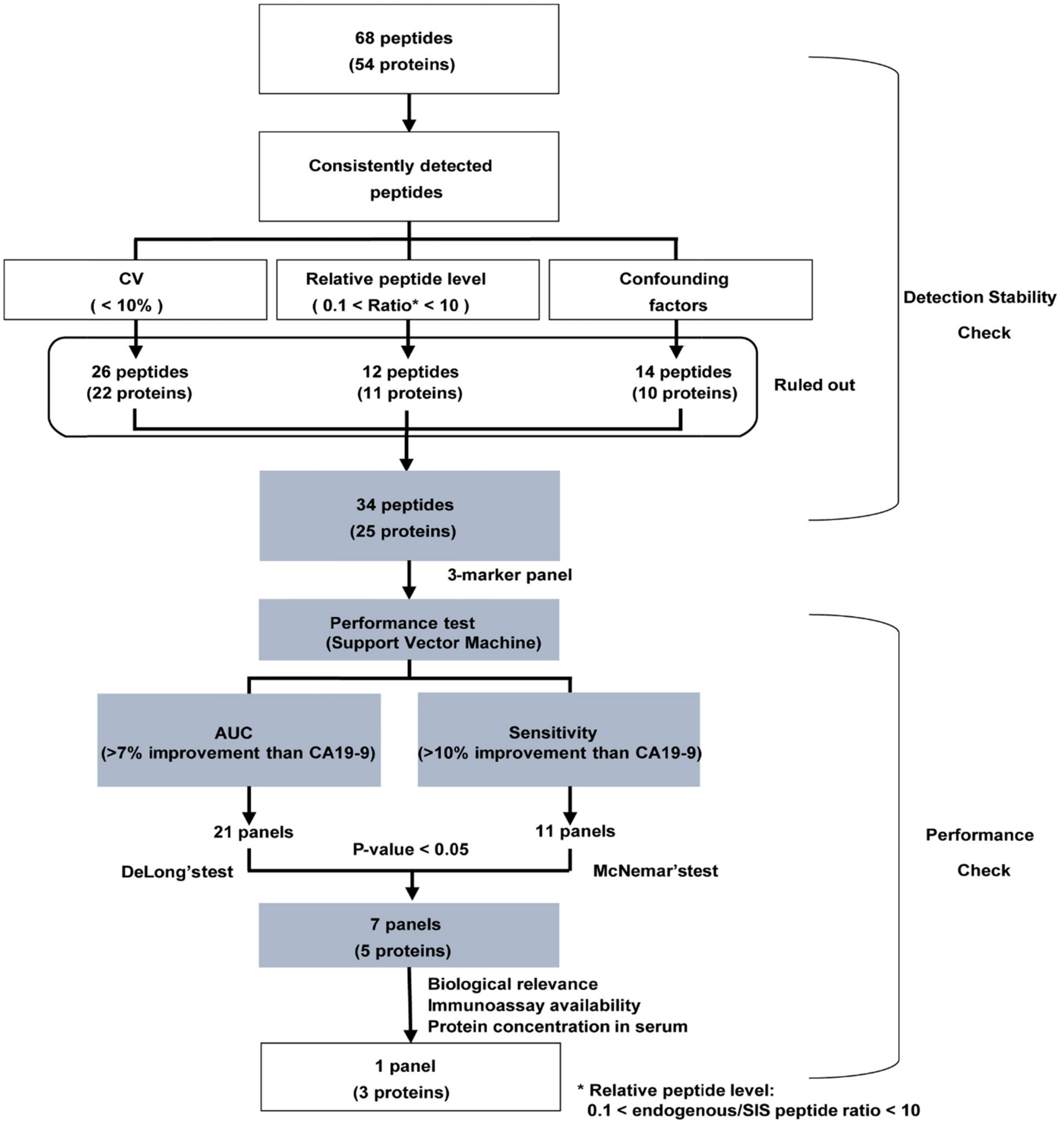

Supplementary Figure 2: Overall construction of the panel. Relative peptide levels (light/heavy ratios) were measured by optimized 68-plex MRM-MS assay. To remove targets with unstable detection, markers with > 10% coefficient of variance (CV), a relative peptide level that was not in the range of 0.1–10, and confounding factors were excluded. All possible combinations of 3-marker panels were tested for their performance and stability by support vector machine (SVM). Three markers were used to construct the most cost-effective and functional panel, based on the result changing with each additional protein. Five-fold cross-validation was performed in the training (n=684) and test sets (n=318). Then, AUC values and sensitivity were measured when the specificity was fixed to 90% in the training set. The AUC values of 18 panels were 7% higher than CA19-9. The sensitivity of 6 panels were 10% higher than that of CA19-9 in the training set. All had *p*-values less than 0.05 by Delong's test. Finally, 5 candidates that satisfied both criteria were selected. The panel with LRG1 and TTR, combined with CA19-9, which had a better AUC value and sensitivity in classifying the participants and greater availability of measurement kits, stability of proteins, and biological relevance, was ultimately selected.

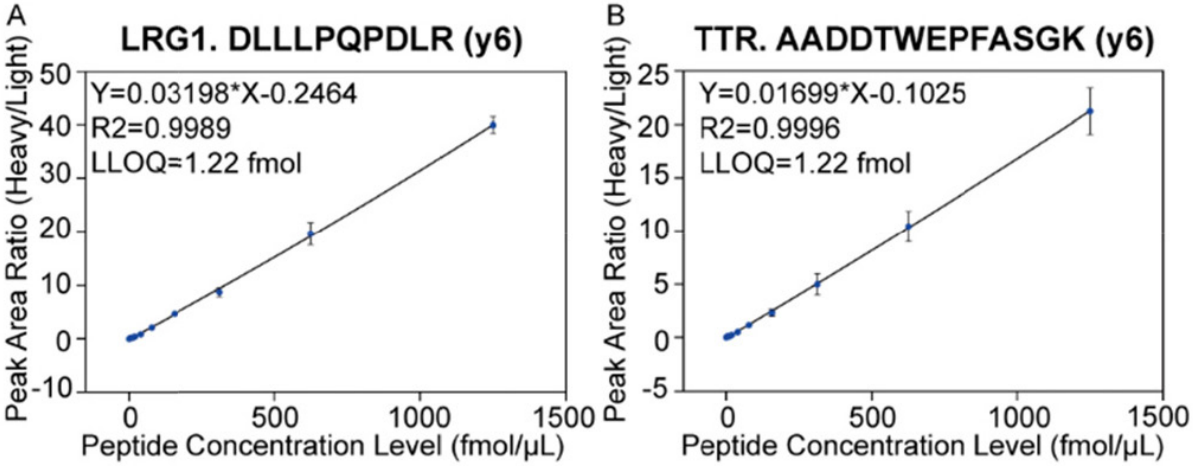

Supplementary Figure 3: Linear response curves of proteins in the triple-marker panel. The title indicates the Uniprot ID name, followed by the peptide sequence and product ion type. Individual concentration points are plotted as the median (shown in blue); error bars are the standard deviation of triplicates. The reverse response curves for the DLLLPQPDLR peptide from LRG1 **(A)** and the AADDTWEPFASGK peptide from TTR **(B)** are shown.

Supplementary Table 1: Demographic characteristics of verification study samples

Institute	SNUH		
**Group**	**PDAC**	**Control**	**PB**
N total	50	50	34
Age mean (SD)	65.72 (9.85)	52.54 (10.27)	64.38 (7.73)
BMI mean (SD)	23.47 (2.54)	23.54* (3.08)	23.94 (2.72)
Sex ratio (Male%)	54	38	67.6
Alcohol ratio (#missing)	26.00 (0)	8.00 (12)	22.58 (3)
Smoking ratio (#missing)	8.00 (0)	8.00 (12)	19.35 (3)
CA19-9 median	168.65	6	8.6
CA19-9 MAD	145.75	3.4	3.75
CA19-9 missing	0	3	0
CA19-9 censor	0	4	0
CEA median	2	1.4	1.4
CEA MAD	0.6	0.5	0.4
CEA missing	2	4	0
CEA censor	0	9	0

SNU, Seoul National University Hospital; MAD, median absolute deviation; CA19-9, carbohydrate antigen 19-9; CEA, carcinoembryonic antigen; PDAC, pancreatic ductal adenocarcinoma; Control, normal and benign group; PB, pancreatic benign (IPMN).
*BMI information of 13 people were missing.

Supplementary Table 2: List of 1000 candidate proteins obtained from four sources

See Supplementary File 1

Supplementary Table 3: The list of 68 interference-free peptides in triplicate analyses with SIS peptides in plasma

See Supplementary File 2

Supplementary Table 4: Composition of sample sets for the training and the test set

Sample	Clinics	Number of samples						
		Total	Training set	Test set				
				Test 1	Test 2	Test 3	Test 4	Test 5
Pancreatic cancer(Stage I/II/III/IV)	Total	396 (20/228/31/117)	316 (16/182/25/93)	80 (4/46/6/24)	50 (4/46/0/0)	80 (4/46/6/24)	80 (4/46/6/24)	29 (3/21/1/4)
	AMC	75 (10/63/1/1)	60 (8/50/1/1)	15 (2/13/0/0)	15 (2/13/0/0)	15 (2/13/0/0)	15 (2/13/0/0)	10 (2/8/0/0)
	NCC	128 (3/27/25/73)	102 (2/22/20/58)	26 (1/5/5/15)	6 (1/5/0/0)	26 (1/5/5/15)	26 (1/5/5/15)	7 (1/3/1/2)
	SMC	96 (1/50/2/43)	76 (1/40/1/34)	20 (0/10/1/9)	10 (0/10/0/0)	20 (0/10/1/9)	20 (0/10/1/9)	5 (0/3/0/2)
	SNUH	50 (3/45/2/0)	40 (2/36/2/0)	10 (1/9/0/0)	10 (1/9/0/0)	10 (1/9/0/0)	10 (1/9/0/0)	1 (0/1/0/0)
	YMC	47 (3/43/1/0)	38 (3/34/1/0)	9 (0/9/0/0)	9 (0/9/0/0)	9 (0/9/0/0)	9 (0/9/0/0)	6 (0/6/0/0)
**Other cancer**	SMC	149	0	0	0	149	0	0
**Pancreatic benign disease**	Total	109	88	21	21		21	21
	AMC	47	33	14	14		14	14
	SMC	30	26	4	4		4	4
	SNUH	5	4	1	1		1	1
	YMC	27	25	2	2		2	2
**Normal**	SNUH	348	280	68	68		0	68
**Total**		**1,002**	**684**	**169**	**139**	**229**	**101**	**118**

The whole sample (n=1,002) was divided into the training (n=684) and the test sets (n=318) for the performance evaluation. Control included normal and pancreatic benign disease (pancreatitis). Overall performance of the panel was determined by Test 1, while the early diagnosis was tested by Test 2. Selectivity was examined by Test 3 in which other cancers were compared with the pancreatic ductal adenocarcinoma. The pancreatic cancer was distinguished from pancreatic benign states by Test 4, and overall performance of the panel on the patients with low level of CA19-9 (<37 U/mL) was evaluated by Test 5. All the pancreatic cancer samples (n=80), the pancreatic benign disease samples (n=21), and the normal samples (n=68) were identical throughout the different test sets. Only early stage samples (n=50) were selected for Test 2, and all stage samples with CA19-9 less than 37 U/mL were used for Test 5.
Test 1. Control (Pancreatic benign disease and normal) vs. Pancreatic ductal adenocarcinoma (All stages).
Test 2. Control (Pancreatic benign disease and normal) vs. Pancreatic ductal adenocarcinoma (stage I & II).
Test 3. Other Cancer (Breast, Colon, Thyroid) vs. Pancreatic ductal adenocarcinoma (All stages).
Test 4. Pancreatic benign disease vs. Pancreatic ductal adenocarcinoma (All stages).
Test 5. Control (Pancreatic benign disease and normal) vs. Pancreatic ductal adenocarcinoma (CA19-9 < 37U/mL).

Supplementary Table 5: The list of panels which outperformed CA19-9 significantly

Model	AUC	$SN\|_{SP=0.9}$	7% higher AUC	10% higher $SN\|_{SP}=0.9$
CA19-9+C1R+TTR	0.963	0.800		
CA19-9+CLU+SERPINC1	0.955	0.867	0	0
CA19-9+SERPINA5+TTR	0.945	0.767		
CA19-9+C5+TTR	0.942	0.900	0	0
CA19-9+LRG1+TTR	0.942	0.867	0	0
CA19-9+C7+TTR	0.938	0.800		
CA19-9+ITIH4+TTR	0.938	0.700		
CA19-9+LRG1+CLU	0.938	0.900	0	0
CA19-9+C7+CLU	0.933	0.900		
CA19-9+CLU+C1R	0.930	0.900		
CA19-9+CPN2+TTR	0.930	0.833		
CA19-9+APOH+TTR	0.925	0.800		
CA19-9+C1R+SEPP1	0.923	0.900		
CA19-9+ITIH2+TTR	0.922	0.700		
CA19-9+ITIH4+CLU	0.920	0.867	0	0
CA19-9+CFI+TTR	0.918	0.733		
CA19-9+LRG1+SEPP1	0.918	0.833		
CA19-9+CFH+TTR	0.907	0.767		
CA19-9+CLU+CFI	0.892	0.800	0	

List of panels with 7% higher AUC values than CA19-9 and those with 10% higher sensitivity when the specificity was 90%. The final 5 panels (CA19-9+CLU+SERPINC1, CA19-9+C5+TTR, CA19-9+LRG1+TTR, CA19-9+ITIH4+CLU, and CA19-9+LRG1+CLU) that satisfied the 2 criteria were considered for further evaluation. All panels were counted based on the proteins that constituted the panel, not the peptides.

Supplementary Table 6: The positive predictive value (PPV) of the triple-marker panel based on the prevalence of pancreatic cancer patients in Korea

	CA19-9	CA19-9 + LRG1 + TTR		
		MRM-MS	Immunoassay 1	Immunoassay 2
**Control vs. PDAC**	0.00083	0.00135 (62.7% ↑)	0.00105 (26.5% ↑)	0.00190 (128.9% ↑)
**Control vs. PDAC Stage I & II**	0.00074	0.00124 (67.6% ↑)	0.00097 (31.08% ↑)	0.00179 (141.9% ↑)
**Other Cancer vs. PDAC**	0.00077	0.00066 (14.3% ↓)	0.00061 (20.8% ↓)	0.00079 (2.6% ↑)
**Pancreatic Benign vs. PDAC**	0.00049	0.00074 (33.8% ↑)	0.00056 (14.3% ↑)	0.00074 (33.8% ↑)
**Control vs. PDAC+**	0.00028	0.00084 (200% ↑)	0.00066 (135.7% ↑)	0.00119 (76.5% ↑)

+ PDAC samples with a normal range of CA19-9 (< 37 U/mL).
Immunoassay 1: LRG1 measured by ELISA, TTR measured by immunoturbidimetric assay (ITA); Immunoassay 2: LRG1 and TTR measured by ELISA.
The positive predictive value (PPV) of the triple-marker panel was calculated by PPV=(Sensitivity×P(D))/(Sensitivity×P(D)+(1-Specificity)(1-P(D))), where P(D) is the prevalence of pancreatic cancer patients in Korea. The comparisons were Control vs. PDAC, Control vs. PDAC Stage I & II, Other Cancer vs. PDAC, Pancreatic Benign vs. PDAC, and Control vs. PDAC with a normal range of CA19-9. The % in the parentheses represents an increase (up arrow) or decrease (down arrow) compared with CA19-9.
