# Supplementary material for: Diagnostic performance enhancement of pancreatic cancer using proteomic multimarker panel
Source: Oncotarget. 2017 Oct 16;8(54):93117–30. doi: 10.18632/oncotarget.21861 (PMC5696248; doi:10.18632/oncotarget.21861)
Supplement: Supplementary file 3 [file oncotarget-08-93117-s003.docx]

**Supplementary Table 3. The list of 68 interference-free peptides in triplicate analyses with SIS peptides in plasma**

| **N.** | **Gene Symbol** | **Uniprot Protein Name** | **Peptide Sequence** | **Precursor Charge** | **Product Charge** | **Fragment Ion** | **Precursor (m/z)** | **Product (m/z)** | **Retention Time (min)** | **Collision energy (V)** | **Result of AuDIT analysis** | |
| --- | --- | --- | --- | --- | --- | --- | --- | --- | --- | --- | --- | --- |
|  |  |  |  |  |  |  |  |  |  |  | ***p*-value** | **CV (%)** |
| 1 | ADIPOQ | Adiponectin | GDIGETGVPGAEGPR | 2 | 1 | y10 | 706.3 | 940.5 | 25.4 | 25.0 | 0.97 | 0.10 |
| 2 | AGT | Angiotensinogen | DPTFIPAPIQAK | 2 | 1 | y7 | 649.4 | 724.4 | 35.2 | 23.0 | 0.61 | 0.04 |
| 3 | ALDH6A1  (MMSA) | Methylmalonate-semialdehyde dehydrogenase | QGIQFYTQLK | 2 | 1 | y7 | 613.3 | 927.5 | 34.9 | 21.7 | 0.00 | 0.16 |
| 4 | APOC1 | Apolipoprotein C-I | EFGNTLEDK | 2 | 1 | y7 | 526.7 | 776.4 | 23.2 | 18.6 | 0.00 | 0.04 |
| 5 | APOC1 | Apolipoprotein C-I | EWFSETFQK | 2 | 1 | y7 | 601.3 | 886.4 | 35.1 | 21.2 | 0.86 | 0.15 |
| 6 | APOH | Beta-2-glycoprotein 1 | VCPFAGILENGAVR | 2 | 1 | y12 | 751.9 | 1243.7 | 39.7 | 26.6 | 0.26 | 0.05 |
| 7 | BTD | Biotinidase | ILSGDPYCEK | 2 | 1 | y8 | 591.3 | 955.4 | 24.3 | 20.9 | 0.48 | 0.01 |
| 8 | BTD | Biotinidase | LSSGLVTAALYGR | 2 | 1 | y8 | 654.4 | 850.5 | 36.8 | 23.1 | 0.63 | 0.04 |
| 9 | C1R | Complement C1r subcomponent | DYFIATCK | 2 | 1 | y6 | 509.2 | 739.4 | 28.7 | 17.9 | 0.54 | 0.04 |
| 10 | C1S | Complement C1s subcomponent | TNFDNDIALVR | 2 | 1 | y9 | 639.3 | 1062.6 | 33.6 | 22.6 | 0.85 | 0.06 |
| 11 | C4BPA | C4b-binding protein alpha chain | TWYPEVPK | 2 | 1 | y6 | 510.3 | 732.4 | 31.5 | 18.0 | 0.33 | 0.04 |
| 12 | C4BPA | C4b-binding protein alpha chain | LSLEIEQLELQR | 2 | 1 | y8 | 735.9 | 1028.6 | 39.6 | 26.1 | 0.86 | 0.15 |
| 13 | C4BPB | C4b-binding protein beta chain | ALLAFQESK | 2 | 1 | y7 | 503.8 | 822.4 | 30.7 | 17.7 | 0.44 | 0.06 |
| 14 | C5 | Complement C5 | NADYSYSVWK | 2 | 1 | y8 | 616.8 | 1047.5 | 30.7 | 21.8 | 0.76 | 0.11 |
| 15 | C5 | Complement C5 | GGSASTWLTAFALR | 2 | 1 | y8 | 719.4 | 977.6 | 45.0 | 25.5 | 0.24 | 0.04 |
| 16 | C6 | Complement component C6 | TLNICEVGTIR | 2 | 1 | y9 | 638.3 | 1061.5 | 33.3 | 22.6 | 0.03 | 0.06 |
| 17 | C7 | Complement component C7 | VLFYVDSEK | 2 | 1 | y7 | 550.3 | 887.4 | 32.1 | 19.4 | 0.50 | 0.08 |
| 18 | CAP1 | Adenylyl cyclase-associated protein 1 | EPAVLELEGK | 2 | 1 | y5 | 542.8 | 575.3 | 31.2 | 19.1 | 0.10 | 0.15 |
| 19 | CDH11 | Cadherin-11 | VLDVNDNAPK | 2 | 1 | y8 | 542.8 | 872.4 | 21.6 | 19.1 | 0.57 | 0.13 |
| 20 | CFH | Complement factor H | GEWVALNPLR | 2 | 1 | y6 | 577.8 | 683.4 | 37.8 | 20.4 | 0.69 | 0.12 |
| 21 | CFH | Complement factor H | TGESVEFVCK | 2 | 1 | y7 | 578.3 | 868.4 | 25.6 | 20.4 | 0.97 | 0.02 |
| 22 | CFI | Complement factor I | VFSLQWGEVK | 2 | 1 | y8 | 596.8 | 946.5 | 38.5 | 21.1 | 0.87 | 0.03 |
| 23 | CLU | Clusterin | TLLSNLEEAK | 2 | 1 | y8 | 559.3 | 903.5 | 33.9 | 19.7 | 0.81 | 0.03 |
| 24 | CLU | Clusterin | ASSIIDELFQDR | 2 | 1 | y8 | 697.4 | 1035.5 | 44.2 | 24.7 | 0.73 | 0.01 |
| 25 | COL4A2 | Collagen alpha-2(IV) chain | GLPGEVLGAQPGPR | 2 | 1 | y7 | 674.4 | 682.4 | 31.5 | 23.9 | 0.00 | 0.23 |
| 26 | CORO1C | Coronin-1C | CDLISIPK | 2 | 1 | y5 | 473.3 | 557.4 | 32.1 | 16.7 | 0.21 | 0.19 |
| 27 | CPN2 | Carboxypeptidase N subunit 2 | LSNNALSGLPQGVFGK | 2 | 1 | y11 | 801.4 | 1102.6 | 38.2 | 28.4 | 0.99 | 0.02 |
| 28 | CTSD | Cathepsin D | VSTLPAITLK | 2 | 1 | y9 | 521.8 | 943.6 | 34.1 | 18.4 | 0.00 | 0.04 |
| 29 | ECM1 | Extracellular matrix protein 1 | ELLALIQLER | 2 | 1 | y8 | 599.4 | 955.6 | 42.7 | 21.2 | 0.98 | 0.08 |
| 30 | FCGBP | IgGFc-binding protein | FAVLQENVAWGNGR | 2 | 1 | y10 | 780.9 | 1130.5 | 36.8 | 27.7 | 0.94 | 0.22 |
| 31 | FSTL1 | Follistatin-related protein 1 | LSFQEFLK | 2 | 1 | y7 | 506.3 | 898.5 | 40.3 | 17.8 | 0.85 | 0.21 |
| 32 | GSTP1 | Glutathione S-transferase P | ASCLYGQLPK | 2 | 1 | y6 | 568.8 | 705.4 | 28.3 | 20.1 | 0.03 | 0.05 |
| 33 | HRG | Histidine-rich glycoprotein | DGYLFQLLR | 2 | 1 | y6 | 562.8 | 789.5 | 45.2 | 19.9 | 0.63 | 0.04 |
| 34 | HSPG2 | Basement membrane-specific heparan sulfate proteoglycan core protein | SPAYTLVWTR | 2 | 1 | y6 | 597.3 | 775.4 | 35.5 | 21.1 | 0.39 | 0.35 |
| 35 | ICAM1 | Intercellular adhesion molecule 1 | LLGIETPLPK | 2 | 1 | y8 | 540.8 | 854.5 | 37.1 | 19.1 | 0.60 | 0.06 |
| 36 | ICAM1 | Intercellular adhesion molecule 1 | VTLNGVPAQPLGPR | 2 | 1 | y8 | 709.9 | 835.5 | 31.7 | 25.1 | 0.28 | 0.03 |
| 37 | IFRD1 | Interferon-related developmental regulator 1 | GLIDLTLDK | 2 | 1 | y7 | 494.3 | 817.5 | 37.6 | 17.4 | 0.00 | 0.21 |
| 38 | IGFBP2 | Insulin-like growth factor-binding protein 2 | LIQGAPTIR | 2 | 1 | y7 | 484.8 | 742.4 | 26.1 | 17.1 | 0.85 | 0.06 |
| 39 | IGFBP3 | Insulin-like growth factor-binding protein 3 | ALAQCAPPPAVCAELVR | 3 | 1 | y9 | 608.3 | 1014.5 | 33.4 | 15.5 | 0.55 | 0.09 |
| 40 | IGFBP3 | Insulin-like growth factor-binding protein 3 | YGQPLPGYTTK | 2 | 1 | y8 | 612.8 | 876.5 | 26.0 | 21.7 | 0.69 | 0.00 |
| 41 | ITIH2 | Inter-alpha-trypsin inhibitor heavy chain H2 | IQPSGGTNINEALLR | 3 | 1 | y6 | 528.3 | 715.4 | 31.8 | 12.3 | 0.56 | 0.26 |
| 42 | ITIH4 | Inter-alpha-trypsin inhibitor heavy chain H4 | LALDNGGLAR | 2 | 1 | y8 | 500.3 | 815.4 | 27.3 | 17.6 | 0.38 | 0.02 |
| 43 | ITIH4 | Inter-alpha-trypsin inhibitor heavy chain H4 | AGFSWIEVTFK | 2 | 1 | y8 | 642.8 | 1009.5 | 45.0 | 22.7 | 0.28 | 0.03 |
| 44 | KLKB1 | Plasma kallikrein | DSVTGTLPK | 2 | 1 | y6 | 459.3 | 616.4 | 22.8 | 16.1 | 0.30 | 0.01 |
| 45 | LDHB | L-lactate dehydrogenase B chain | IVVVTAGVR | 2 | 1 | y7 | 457.3 | 701.4 | 26.9 | 16.1 | 0.03 | 0.14 |
| 46 | LDHB | L-lactate dehydrogenase B chain | GLTSVINQK | 2 | 1 | y7 | 480.3 | 789.4 | 25.7 | 16.9 | 0.80 | 0.05 |
| 47 | LRG1 | Leucine-rich alpha-2-glycoprotein | DLLLPQPDLR | 2 | 1 | y6 | 590.3 | 725.4 | 38.0 | 20.8 | 0.33 | 0.03 |
| 48 | LRG1 | Leucine-rich alpha-2-glycoprotein | VAAGAFQGLR | 2 | 1 | y8 | 495.3 | 819.4 | 27.7 | 17.4 | 0.44 | 0.02 |
| 49 | MBL2 | Mannose-binding protein C | FQASVATPR | 2 | 1 | y7 | 488.8 | 701.4 | 22.9 | 17.2 | 0.26 | 0.04 |
| 50 | P4HB | Protein disulfide-isomerase | ALAPEYAK | 2 | 1 | y6 | 431.7 | 678.3 | 18.9 | 15.2 | 0.85 | 0.11 |
| 51 | PDCD4 | Programmed cell death protein 4 | GDSVSDSGSDALR | 2 | 1 | y9 | 633.3 | 907.4 | 19.1 | 22.4 | 0.00 | 0.23 |
| 52 | PKM2 | Pyruvate kinase | CDENILWLDYK | 2 | 1 | y6 | 734.8 | 837.5 | 40.1 | 26.0 | 0.18 | 0.11 |
| 53 | PPBP | Platelet basic protein | NIQSLEVIGK | 2 | 1 | y8 | 550.8 | 873.5 | 24.4 | 19.4 | 0.41 | 0.00 |
| 54 | PPBP | Platelet basic protein | ICLDPDAPR | 2 | 1 | y7 | 528.8 | 783.4 | 27.1 | 18.6 | 0.00 | 0.06 |
| 55 | PROS1 | Vitamin K-dependent protein S | NNLELSTPLK | 2 | 1 | y7 | 564.8 | 787.5 | 30.4 | 19.9 | 0.39 | 0.03 |
| 56 | PTPRJ | Receptor-type tyrosine-protein phosphatase eta | DTEVLLVGLEPGTR | 2 | 1 | y9 | 749.9 | 941.5 | 40.0 | 26.6 | 0.11 | 0.28 |
| 57 | SEPP1 | Selenoprotein P | CINQLLCK | 2 | 1 | y6 | 524.8 | 775.4 | 28.1 | 18.5 | 0.83 | 0.02 |
| 58 | SERPINA5(IPSP) | Plasma serine protease inhibitor | GFQQLLQELNQPR | 2 | 1 | y8 | 785.9 | 997.5 | 43.0 | 27.9 | 0.80 | 0.10 |
| 59 | SERPINC1 | Antithrombin-III | VAEGTQVLELPFK | 2 | 1 | y7 | 715.9 | 845.5 | 39.5 | 25.3 | 0.98 | 0.07 |
| 60 | SERPINC1 | Antithrombin-III | IEDGFSLK | 2 | 1 | y7 | 454.7 | 795.4 | 29.0 | 16.0 | 0.30 | 0.03 |
| 61 | SFTPB | Pulmonary surfactant-associated protein B | FLEQECNVLPLK | 2 | 1 | y10 | 745.4 | 1229.6 | 36.3 | 26.4 | 0.35 | 0.20 |
| 62 | SOD3 | Extracellular superoxide dismutase | VTGVVLFR | 2 | 1 | y7 | 445.8 | 791.5 | 33.3 | 15.7 | 0.40 | 0.06 |
| 63 | SPARC | SPARC | NVLVTLYER | 2 | 1 | y7 | 553.8 | 893.5 | 34.3 | 19.5 | 0.92 | 0.14 |
| 64 | THBS1 | Thrombospondin-1 | GFLLLASLR | 2 | 1 | y6 | 495.3 | 672.4 | 44.1 | 17.4 | 1.00 | 0.27 |
| 65 | THBS1 | Thrombospondin-1 | TIVTTLQDSIR | 2 | 1 | y9 | 623.9 | 1032.6 | 36.0 | 22.0 | 0.93 | 0.12 |
| 66 | TTR | Transthyretin | AADDTWEPFASGK | 2 | 1 | y8 | 697.8 | 921.4 | 33.3 | 24.7 | 1.00 | 0.10 |
| 67 | TXN | Thioredoxin | CMPTFQFFK | 2 | 1 | y7 | 603.3 | 914.5 | 41.1 | 21.3 | 0.84 | 0.16 |
| 68 | VIM | Vimentin | ILLAELEQLK | 2 | 1 | y8 | 585.4 | 943.5 | 40.6 | 20.7 | 0.77 | 0.07 |
